# *In vivo* human lower limb muscle architecture dataset obtained using diffusion tensor imaging

**DOI:** 10.1371/journal.pone.0223531

**Published:** 2019-10-15

**Authors:** James P. Charles, Felipe Suntaxi, William J. Anderst

**Affiliations:** 1 Evolutionary Morphology and Biomechanics Lab, Institute of Aging and Chronic Disease, University of Liverpool, Liverpool, United Kingdom; 2 Biodynamics Lab, Department of Orthopaedic Surgery, University of Pittsburgh, Pennsylvania, United States of America; New York Institute of Technology, UNITED STATES

## Abstract

‘Gold standard’ reference sets of human muscle architecture are based on elderly cadaveric specimens, which are unlikely to be representative of a large proportion of the human population. This is important for musculoskeletal modeling, where the muscle force-generating properties of generic models are defined by these data but may not be valid when applied to models of young, healthy individuals. Obtaining individualized muscle architecture data *in vivo* is difficult, however diffusion tensor magnetic resonance imaging (DTI) has recently emerged as a valid method of achieving this. DTI was used here to provide an architecture data set of 20 lower limb muscles from 10 healthy adults, including muscle fiber lengths, which are important inputs for Hill-type muscle models commonly used in musculoskeletal modeling. Maximum isometric force and muscle fiber lengths were found not to scale with subject anthropometry, suggesting that these factors may be difficult to predict using scaling or optimization algorithms. These data also highlight the high level of anatomical variation that exists between individuals in terms of lower limb muscle architecture, which supports the need of incorporating subject-specific force-generating properties into musculoskeletal models to optimize their accuracy for clinical evaluation.

## Introduction

The musculoskeletal architecture (*i*.*e*. the macroscopic arrangement of muscle fibers [1]) of the human lower limb has been well defined, with several extensive data sets published [2, 3]. However, these “gold standard” reference data sets are based on elderly cadaveric specimens, which for various reasons, such as possible changes in muscle architecture due to aging [4], are unlikely to be representative of young, active and healthy adults [5]. These differences have been highlighted in regards to muscle volumes [5, 6], although the extent of variation in muscle architecture properties such as muscle fiber length, pennation angle and maximum isometric force is largely unknown. This is particularly important in the context of musculoskeletal modeling using dimensionless Hill-type muscle models [7], which are defined by these properties. Importantly, various sensitivity analyses have shown that these models are particularly sensitive to even small changes in muscle fiber and tendon slack lengths in particular [8–14]. Furthermore, how well these parameters scale with respect to body anthropometric factors such as body or limb mass has also not been reported in detail, although it has been suggested that fiber lengths may not scale particularly strongly with bone length [15]. While muscle architecture parameters can be estimated through optimization [16–19], directly measuring these *in vivo* may improve the accuracy of computational models.

Using a previously established framework of diffusion tensor imaging (DTI) and fiber tractography, in combination with other magnetic resonance imaging sequences [20], this study aims to build on previous literature and provide a detailed human lower limb *in vivo* muscle architecture data set. This will also highlight the level of inter-subject variability in muscle architecture parameters which exists in young healthy adults, as well as the scaling relationships between muscle architecture and body proportions.

## Methods

For the present study, data were gathered from 20 muscles in the right lower limbs of 10 healthy, non-professionally athletically trained adults (5 males, 5 females; Age- 27 ± 4 years. Body mass- 76 ± 12 kg; Table 1), who signed informed consent documents prior to participating in this IRB-approved study. The muscles analyzed were classified into 5 distinct functional groups based on major functions (Table 2), which were based on previous human muscle architecture studies [3]. Muscle fiber length, fiber pennation angle and muscle volumes were estimated from each of these muscles, using a validated framework of magnetic resonance imaging (MRI) and DTI [20]; see below).

**Table 1:** Study participant information. Thigh length- the distance between the most proximal aspect of the greater trochanter of the femur, and the most distal aspect of the lateral femoral condyle. Leg length- the distance between the tibial plateau and the center of the ankle (tibiotalar) joint. L_L_—Total lower limb length. V_LM_—Total lower limb muscle volume (sum of volumes of the studied muscles). V_L_—Total lower limb volume (sum of muscle volumes plus fat, fascia and skin).

Subject Number	Sex	Age (years)	Body Mass (kg)	Height (cm)	BMI (kgm^-2^)	Thigh length (cm)	Lower leg length (cm)	L_L_ (cm)	V_LM_ (cm^3^)	V_L_ (cm^3^)
**01**	Male	23	90.7	182	27.4	46.3	39.3	85.6	6547	11620
**02**	Male	26	82.1	173	27.4	42.3	38.0	80.3	5128	11971
**03**	Male	29	81.1	182	24.1	45.3	39.4	84.7	5040	7066
**04**	Female	26	71.2	162	27.1	40.7	37.7	78.4	4209	12173
**05**	Female	23	59.8	170	20.7	41.7	38.3	80.0	3989	9450
**06**	Female	35	80.2	169	28.1	42.4	35.6	78.0	4386	10462
**07**	Female	25	80.7	168	28.6	42.1	34.7	76.8	3153	12853
**08**	Female	26	40.6	162	17.8	39.0	33.6	72.6	2955	6101
**09**	Male	26	84.8	187	24.5	46.3	42.7	89.0	6119	11517
**10**	Male	34	82.5	192	22.4	45.7	42.7	88.4	4655	9008

**Table 2:** Muscle functional group classifications, based on those from Ward et al., [3].

Functional group	Muscle	Abbreviation
**Hip adductors**	Adductor magnus	AM
	Adductor longus	AL
	Adductor brevis	AB
	Gracilis	GRA
**Knee flexors**	Semimembranosus	SM
	Semitendinosus	ST
	Biceps femoris (long head)	BFL
	Biceps femoris (short head)	BFS
	Popliteus	POP
	Sartorius	SAR
**Knee extensors**	Rectus femoris	RF
	Vastus lateralis	VL
	Vastus medialis	VM
	Vastus intermedius	VI
**Ankle dorsiflexors**	Tibialis anterior	TA
	Extensor digitorum longus	EDL
	Extensor hallucis longus	EHL
**Ankle plantarflexors**	Medial gastrocnemius	MG
	Lateral gastrocnemius	LG
	Soleus	SOL

### MRI and DTI acquisition

**Fig 1:**
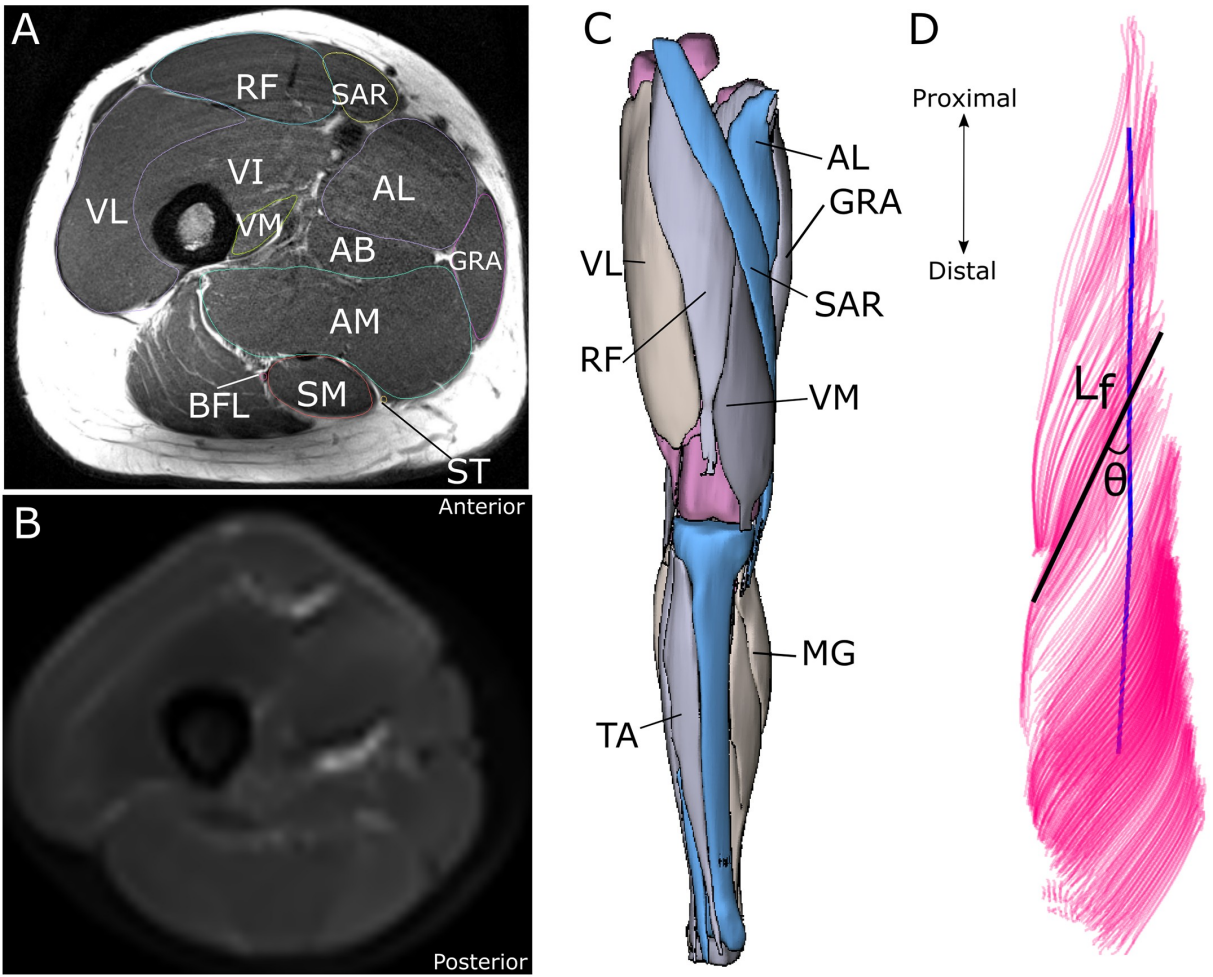
Representative T1-weighted MR anatomical image (A) and diffusion tensor image (B) of the thigh segment of one subject. Muscles and bones were digitally segmented from the T1 images to create 3D representations of the lower limbs (C) (for muscle abbreviation definitions, see Table 2). Muscle fascicles (fibers) were tracked from the diffusion weighted MR images (D). From these 3D point cloud-based models, it was possible to measure fiber length (L_f_) and surface fiber pennation angle (θ, angle of the fibers relative to the muscle’s line of action (blue line)).

All MR images were acquired from the iliac crest to the ankle joint using a 3 T scanner (Biograph mMR, Siemens, Munich, Germany), with each subject in a supine position and with the lower limbs in the anatomical position. Imaging consisted of two sequences (Fig 1A and 1B): T1-weighted anatomical turbo spin echo (TSE) (voxel size 0.47×0.47×6.5 mm^3^, repetition time [TR]—650 ms, echo time [TE]—23 ms, number of slices—35 per segment, number of signal averages (NSA)—1, acceleration factor—2), and diffusion-weighted single-shot dual-refocusing spin-echo planar (voxel size 2.96×2.96×6.5 mm^3^, TR/TE 7900/65 ms, 12 direction diffusion gradients, *b* value—0 & 400 s/mm^2^, strong fat suppression—spectral attenuated inversion recovery [SPAIR], number of slices—35 per segment, NSA—2, acceleration factor—2, bandwidth—2440 Hz/pixel). Advanced B_0_ shimming was done for each segment to reduce spatial distortion and minimize the residual fat chemical shift in the diffusion-weighted images, in the phase-encoding direction (anterior to posterior). For each subject, images were acquired in an axial slice orientation, and repeated for a total of five to six segments, which were merged during post-processing using the Stitching plugin for Fiji/ImageJ [21, 22]. Total image acquisition time was ~37mins per subject.

The T1- weighted MR images were digitally segmented in Mimics (Materialise, Leuven, Belgium) to create three-dimensional meshes of each muscle (Fig 1C), which allowed for the determination of individual muscle (belly) volumes (mm^3^).

### DTI pre-processing and fiber tractography

The diffusion tensor images were pre-processed to reduce image artefacts and improve signal to noise ratio. To reduce image artifacts caused by the possible motion of the subjects or spatial distortion (eddy currents and/or magnetic field inhomogeneity), each diffusion-weighted image was registered to the non-diffusion weighted image (with b value 0) using an affine transformation in DTI-studio [23].

To reduce the signal to noise ratio of the images, a Rician noise suppression algorithm was applied to the DTI images [24] in MedINRIA (www.med.inria.fr), where the diffusion tensors for each subject were estimated and smoothed. Manual thresholding removed background pixels from the tensor estimation. Muscle fascicles for each muscle were estimated from these tensors with tractography in Camino software [25], producing fiber tracts, from regions of interest (ROIs) drawn based on the anatomical T1 MR images (Fig 1D). These tracts were tracked bidirectionally (step size 1mm) from seeding regions of interest (ROIs) and continued until terminated based on defined fiber curvature stopping criteria (angle change >10 degrees per 5mm). These tractography settings were kept consistent between muscles and subjects.

While muscle fibers may not necessarily extend the entire length of a muscle fascicle (bundles of ~5–10 fibers) and may instead be connected in series, it has been shown that fibers in this arrangement may be activated simultaneously to act like a single fiber [26]. It was therefore assumed here that fiber lengths are functionally equivalent to fascicle lengths, and these terms are used interchangeably. Based on this assumption, custom MATLAB code (available at www.figshare.com—DOI: 10.6084/m9.figshare.9906266) was used to measure L_f_ from these fiber tracts (equivalent to muscle fascicles), and values reported here are means of the entire range of fiber tract lengths throughout each muscle [20]. This is standard practice when measuring muscle architecture for inputs into Hill type muscle models [3, 27], and in this context has been shown to estimate L_f_ to an average accuracy of <1 ± 7 mm [20].

The pennation angle of these fibers was measured here as the angle of the fibers relative to the muscle’s line of action. Each muscles’ line of action was estimated using the “fit centerline” function on each 3D muscle mesh in Mimics (from the T1-weighted MR images), which estimates a line through the axial centroids of each mesh, and therefore accounts for their often-curved shapes. The assumption that this line is equivalent to an anatomical line of action has been reported previously [6]. Five superficial (2D) pennation angle measurements were manually recorded at proximal, middle and distal areas of each muscle using ImageJ [28] to obtain a representative mean value. This is also standard practice for estimating this parameter for musculoskeletal models, and has been shown to estimate surface pennation angles to an average accuracy of 4 ± 1° [20].

All these methods were performed by the same researcher for each subject, ensuring consistency in the reported muscle architecture data.

### Predicting optimal fiber lengths

A previously recognized limitation of measuring fiber lengths from diffusion tensor images is that estimates of optimal fiber lengths (an important input to musculoskeletal models) are not obtainable using this method alone. This is because sarcomere lengths, which are normalized to a standardized optimal resting sarcomere length to estimate optimal fiber lengths, are not directly measurable from the tracked fibers. Therefore, optimal fiber lengths were estimated using sarcomere lengths reported in [3], using the following equation [29]:

$$Lf=Lf\prime (\frac{2.7\mu m}{Ls}),$$

where L_f_ is optimal fiber length, L_f_’ is raw fiber length (measured from DTI), L_s_ is sarcomere length, and 2.7μm is a generic value for optimal sarcomere length [29]. L_s_ values were obtained from Ward et al. [3], who measured L_s_ in fixed muscles dissected from limbs with most joints (other than the ankle joint) in the anatomical position, as in the present study.

These parameters were then used to calculate physiological cross-sectional area (PCSA, mm^2^), a major determinant of muscle force output, using the equation (from [30]):

$$\text{PCSA}=({\text{V}}_{\text{m}}*\text{cos}\theta )/({\text{L}}_{\text{f}}),$$

where V_m_ is muscle (belly) volume (mm^3^), L_f_ is optimal muscle fiber length (mm), θ is muscle fiber pennation angle. To estimate maximum isometric force (an important input parameter for musculoskeletal models, F_max_), individual PCSA values were multiplied by the isometric stress of skeletal muscle (or specific tension; 0.3Nmm^-2^; [7]). The use of this generic value for isometric stress is well established within musculoskeletal modeling research [13, 31], and has been shown to be independent on body size and conserved within vertebrate phylogeny [32]. Given that estimating this value for each individual muscle of the lower limb was out of the scope of this study, it was assumed here to be constant across all muscles. However, it is recognized that in reality this may not be the case, with a wide range of values (0.04–0.6 Nmm^2^) reported in the literature within human lower limb muscles, depending on function or fiber type [33, 34].

Specializations in muscle architecture parameters within functional groups (i.e. certain muscle functional groups, such as knee extensors, show broadly similar muscle fiber orientations and by extension functional capabilities) have been demonstrated previously in the vertebrate musculoskeletal system [27, 30, 35–41]. Therefore, muscle architecture data obtained here for each muscle were averaged over each functional group within each individual, as well as within the grouped mean values (Tables 1, 2 & S1–S10 Tables). This gives a general insight into the degree of these muscle functional group specializations within the lower limbs of the individuals in this study, and also allows comparisons to similar functional group averages in previous architecture data sets (3).

How these muscle architecture variables scale with body mass, height, total limb volume (V_L_) and limb length (L_L_) across the different individuals in our study population was tested using linear regression in GraphPad Prism (La Jolla, California, USA; www.graphpad.com). Limb length was defined as the length from the most proximal aspect of the greater trochanter of the femur, to the most distal aspect of the lateral malleolus of the fibula.

## Results

The mean (± standard deviation) *in vivo* architecture properties for 20 lower limb muscles as measured from DTI and T1 MRI sequences across 10 individuals were determined (Table 3). Muscle architecture data for individual subjects are listed in S1–S10 Tables.

**Table 3:** Mean (± standard deviations) architecture properties of 20 lower limb muscles from 10 individuals (5 males, 5 females; Age- 27.3 ± 3.95 years. Body mass- 76 ± 12.5 kg), plus functional group means. L_F_:L_m_- Muscle fiber length muscle length ratio. PCSA- Physiological cross-sectional area. F_max_- estimated maximum isometric force. Sarcomere lengths used to estimate optimal fiber lengths were sourced from Ward et al., [3].

Muscle	Muscle Volume (cm^3^)	Muscle Length (mm)	Optimal fiber length (mm)	L_f_:L_m_	Pennation angle (°)	PCSA (mm^2^)	F_max_ (N)	F_max_ (%BW)
**Adductor magnus**	567 ± 186	303 ± 31	231 ± 61	0.74 ± 0.17	12 ± 3	2524 ± 859	757 ± 258	106 ± 44
**Adductor longus**	159 ± 56	219 ± 27	110 ± 27	0.51 ± 0.14	12 ± 2	1470 ± 528	441 ± 158	60 ± 20
**Adductor brevis**	93 ± 21	151 ± 28	76 ± 22	0.51 ± 0.15	11 ± 2	1268 ± 369	380 ± 111	53 ± 17
**Gracilis**	91 ± 32	343 ± 28	173 ± 56	0.50 ± 0.14	6 ± 2	531 ± 104	159 ± 31	23 ± 8
**Semimembranosus**	244 ± 57	272 ± 29	158 ± 43	0.58 ± 0.14	12 ± 3	1561 ± 368	468 ± 110	64 ± 14
**Semitendinosus**	186 ± 55	324 ± 26	183 ± 45	0.57 ± 0.14	8 ± 2	1073 ± 438	322 ± 131	43 ± 15
**Biceps femoris- long head**	194 ± 55	261 ± 26	204 ± 38	0.79 ± 0.15	11 ± 5	998 ± 502	299 ± 151	39 ± 15
**Biceps femoris- short head**	92 ± 22	279 ± 39	109 ± 21	0.40 ± 0.13	9 ± 1	849 ± 220	255 ± 66	35 ± 9
**Popliteus**	15 ± 5	98 ± 22	74 ± 14	0.78 ± 0.17	8 ± 1	202 ± 76	60 ± 23	8 ± 2
**Sartorius**	143 ± 38	504 ± 48	408 ± 30	0.85 ± <0.01	N/A	349 ± 85	105 ± 26	14 ± 2
**Rectus femoris**	249 ± 65	323 ± 28	142 ± 43	0.44 ± 0.13	8 ± 1	1853 ± 591	556 ± 177	78 ± 34
**Vastus lateralis**	606 ± 151	335 ± 21	196 ± 42	0.59 ± 0.14	15 ± 4	3206 ± 1559	962 ± 468	129 ± 51
**Vastus medialis**	415 ± 115	336 ± 44	159 ± 39	0.48 ± 0.14	14 ± 3	2707 ± 1119	812 ± 336	110 ± 40
**Vastus intermedius**	521 ± 124	353 ± 28	181 ± 40	0.51 ± 0.11	11 ± 4	2938 ± 926	881 ± 278	122 ± 41
**Tibialis anterior**	129 ± 22	300 ± 43	137 ± 26	0.46 ± 0.08	7 ± 2	955 ± 197	286 ± 59	39 ± 10
**Extensor digitorum longus**	76 ± 17	348 ± 29	138 ± 26	0.40 ± 0.07	7 ± 2	570 ± 185	171 ± 56	24 ± 9
**Extensor hallucis longus**	21 ± 7	238 ± 46	106 ± 24	0.45 ± 0.09	7 ± 2	196 ± 78	59 ± 23	8 ± 3
**Medial gastrocnemius**	230 ± 48	254 ± 24	97 ± 22	0.38 ± 0.07	10 ± 4	2371 ± 433	711 ± 130	97 ± 15
**Lateral gastrocnemius**	128 ± 35	240 ± 45	122 ± 44	0.51 ± 0.15	9 ± 3	1159 ± 483	348 ± 145	47 ± 18
**Soleus**	461 ± 108	349 ± 28	146 ± 32	0.42 ± 0.09	12 ± 2	3226 ± 1042	968 ± 313	130 ± 35
**Hip adductors**	**227 ± 74**	**254 ± 28**	**147 ± 41**	**0.57 ± 0.15**	**11 ± 2**	**1448 ± 465**	**434 ± 140**	**60 ± 22**
**Knee flexors**	**146 ± 39**	**290 ± 32**	**189 ± 32**	**0.66 ± 0.12**	**8 ± 2**	**839 ± 281**	**252 ± 84**	**34 ± 10**
**Knee extensors**	**448 ± 114**	**337 ± 30**	**170 ± 41**	**0.51 ± 0.13**	**12 ± 3**	**2676 ± 1049**	**803 ± 315**	**110 ± 41**
**Ankle dorsiflexors**	**75 ± 15**	**296 ± 39**	**127 ± 25**	**0.44 ± 0.08**	**7 ± 2**	**574 ± 154**	**172 ± 46**	**24 ± 7**
**Ankle plantarflexors**	**273 ± 64**	**281 ± 32**	**122 ± 32**	**0.44 ± 0.10**	**10 ± 3**	**2252 ± 652**	**676 ± 196**	**91 ± 23**

On average, the muscles in the lower limb with the largest PCSA were vastus lateralis (3206 ± 1559 mm^2^), vastus intermedius (2938 ± 926 mm^2^), and soleus (3226 ± 1042 mm^2^). Muscles with the smallest PCSA across all subjects were extensor hallucis longus (196 ± 78 mm^2^), popliteus (202 ± 76 mm^2^) and sartorius (333 ± 84 mm^2^).

The muscles with the longest L_f_ were on average; sartorius (408 ± 30 mm), adductor magnus (231 ± 61mm) and biceps femoris (long head) (204 ± 38mm). The muscles with the shortest L_f_ were popliteus (74 ± 14 mm), adductor brevis (76 ± 22 mm) and medial gastrocnemius (97 ± 22 mm). When fiber length was normalized to muscle length (L_f_:L_m_), the muscles with the largest L_f_:L_m_ ratios were; sartorius (0.85 ± <0.01), biceps femoris long head (0.74 ± 0.15), popliteus (0.78 ± 0.17), adductor magnus (0.74 ± 0.17) and vastus lateralis (0.59 ± 0.14). The muscles with the smallest L_f_:L_m_ were soleus (0.32 ± 0.07), medial gastrocnemius (0.39 ± 0.08) and rectus femoris (0.39 ± 0.12).

**Fig 2:**
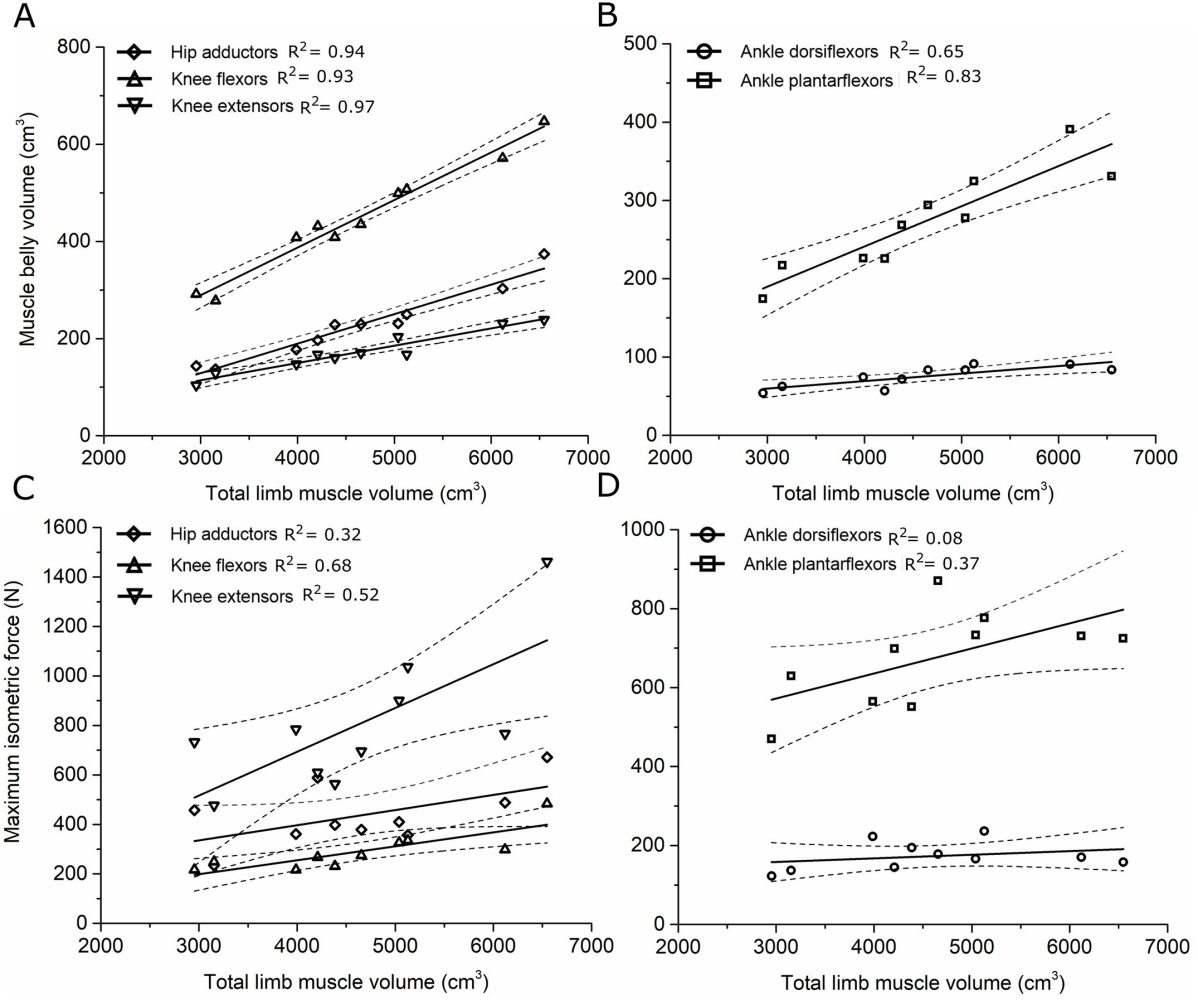
The scaling relationships between: Individual total limb muscle volume and total muscle belly volume in the (A) hip adductors, knee flexors and knee extensors; (B) ankle dorsiflexors and ankle plantarflexors; and maximum isometric force in the (C) hip adductors, knee flexors and knee extensors; (D) ankle dorsiflexors and ankle plantarflexors. Dotted lines represent ±1 standard error mean.

**Fig 3:**
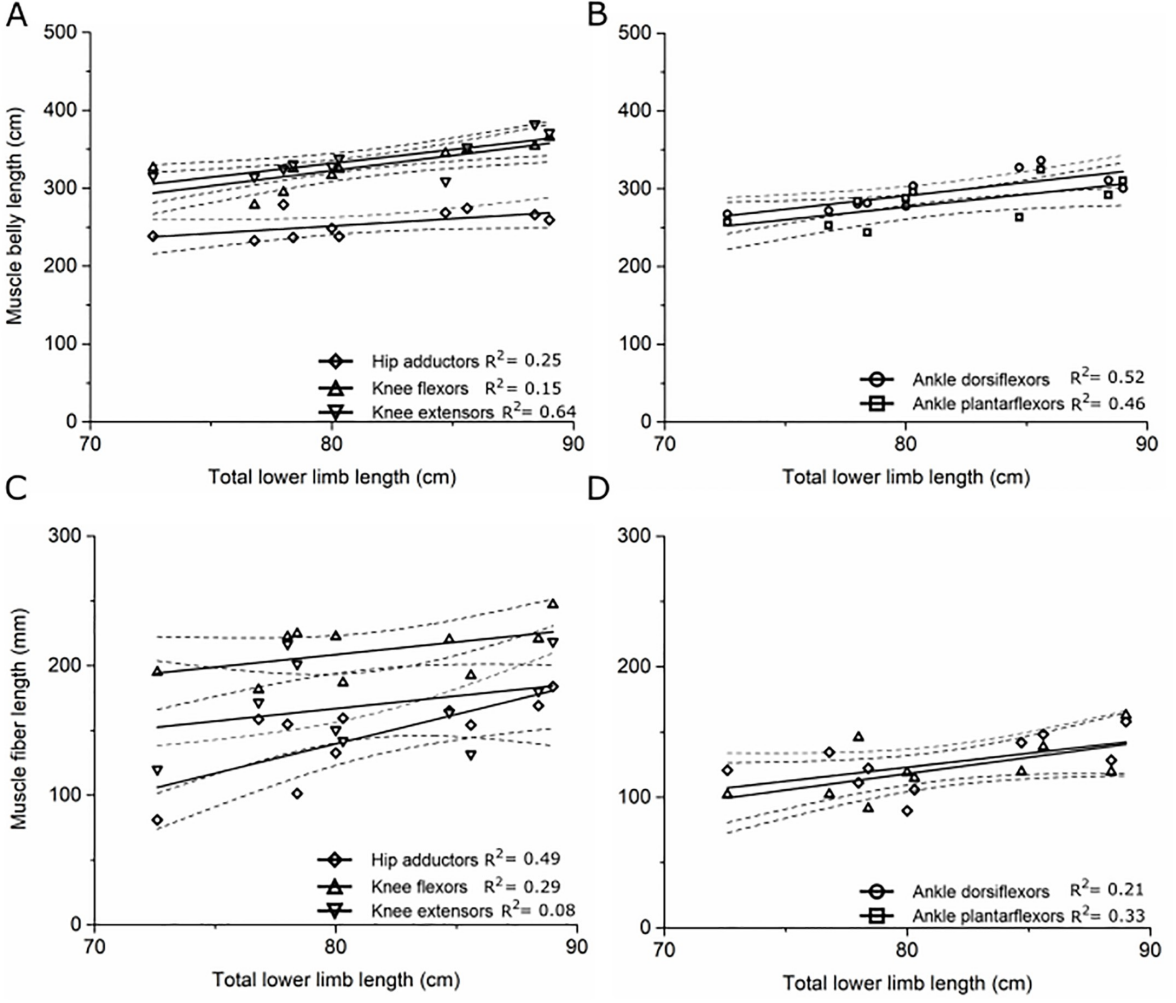
The scaling relationships between: Individual limb length and muscle belly length in the (A) hip adductors, knee flexors and knee extensors; (B) ankle dorsiflexors and ankle plantarflexors; and muscle fiber length in the (C) hip adductors, knee flexors and knee extensors; (D) ankle dorsiflexors and ankle plantarflexors. Dotted lines represent ±1 standard error mean.

The degree to which muscle volume, fiber length and maximum isometric force scaled with subject total limb volume (V_LM_) and limb length (L_L_) varied considerably between the muscle functional groups (Figs 2 and 3; S11 Table). The mean volume of the muscle groups scaled strongly with V_LM_ (Fig 2A & 2B), although only the knee flexors and knee extensors showed statistically significant scaling relationship between F_max_ and M_L_ (R^2^ > 0.5, p < 0.05; Fig 3C, S11 Table). Muscle belly length scaled with L_L_ (Fig 3A & 3B), however L_f_ did not scale particularly strongly with L_L_ in any functional group, with the hip adductors showing the strongest and only statistically significant correlation (R^2^ = 0.49, p = 0.02; Fig 3C, S11 Table).

## Discussion

This study used a validated technique to generate an extensive data set of *in vivo* human lower limb muscle architecture data exclusively from MR images of 10 young healthy individuals. These data define muscle volume, length, optimal fiber length, fiber pennation angle, PCSA and maximum isometric force. The technique of using DTI and muscle fiber tractography to gather detailed muscle architecture data has been previously described and shown to be valid and repeatable [20, 42–56]. In a study into the validity of the technique for gathering muscle architecture specifically for musculoskeletal models, Charles et al., [20] found that DTI can replicate muscle masses, fiber lengths and PCSA within 4%, 1% and 6% of the corresponding variables measured from manual dissections, respectively. The accuracy of this method raised confidence in our ability to generate an accurate and reliable data set of *in vivo* lower limb muscle architecture from a population of young healthy adults (Table 2; S1–S10 Tables). This dataset builds on previous attempts to quantify lower limb muscle anatomy with MRI [6], by focusing on gathering the architecture data necessary to inform musculoskeletal models and simulations.

The degree to which these muscle architecture data scale with anthropometric parameters such as limb length, limb volume and body mass, as well as age, within muscle functional groups could indicate the necessity for gathering such an extensive *in vivo* muscle architecture data set on young, healthy individuals. Muscle belly volume and muscle belly length both scaled reasonably well with limb mass and limb length, respectively, within most functional groups. These scaling relationships are particularly apparent in the ankle plantarflexor muscles, where F_max_ and muscle volume show strong correlations with subject height, body mass, limb volume and limb length. These results agree with those of Handsfield et al., [6], who reported similarly strong scaling relationships between muscle belly volume/length and limb length and body mass. However, these data show that muscle fiber length does not scale well with limb length in any muscle functional group, which agrees with previous studies [15].

In the context of musculoskeletal modeling, this suggests that the relationship between muscle fiber length and limb length may not necessarily be accurately predicted using scaling or optimization algorithms and could be more complex than other muscle architecture variables. So, while anthropometric scaling can be used to estimate gross anatomical properties such as muscle volume and length, subject-specific imaging of lower limb anatomy is likely necessary to accurately estimate more complex muscle architecture parameters such as muscle fiber lengths, particularly for use in musculoskeletal modeling. The lack of direct correlation between fiber lengths and limb lengths could be explained by inter-subject variation in the length of the external tendinous portion of the musculotendon unit, which has been shown to be related to joint range of motion, particularly in the distal muscle groups of the lower limb [57].

Gathering subject-specific data is further justified by the differences between these data and previously published cadaveric architecture data, such as that described by Ward et al., [3]. While the general trends in mean architecture properties in our data closely followed those previously described (with many of the same muscles having large PCSA values and long optimal fiber lengths), many differences in absolute values can be seen (see supplementary information for more details). Given the anatomical variation seen within our data set, these differences are most likely due to the variable degrees of muscle architecture scaling between muscle functional groups, as well as the potential effects of ageing. In a study into the kinematic and kinetic effects of ageing, DeVita and Hortobagyi [58] suggested that ageing results in a redistribution of joint and muscle torques throughout the lower limb, with relatively lower ankle but larger hip joint torques and muscle power in elderly compared to younger individuals. While likely associated with changes in gait kinematics with advancing age, this alteration of joint torques could also arise as a result of changes in complex muscle architecture (i.e. reductions in L_f_:L_m_), which were shown here through comparisons to cadaveric muscle architecture and were particularly evident in distal muscles.

This supports the accuracy of our data, however as muscle volumes and fiber lengths have only been predicted to decrease 25% and 10% respectively due to ageing effects [4], anatomical variation, in addition to effects of formalin fixation or possible pathologies in cadaveric specimens in previous dissection studies [3], is likely another significant reason for differences between these data and cadaveric data. This high level of anatomical variation also supports the potential need for subject-specific musculoskeletal modeling for clinical evaluation. Individualized models have become more common [11, 12, 59–62], and with novel methods of gathering in vivo muscle architecture, these models could potentially provide more accurate and reliable estimates of muscle function compared to generic or scaled generic models.

### Limitations

While this method of gathering *in vivo* muscle architecture is becoming more common [20, 53–56], there are still some limitations that must be overcome before widespread use in the musculoskeletal modeling and simulation community. Many of these limitations, such as assumptions in how pennation angles and fiber lengths were estimated, are similar to those discussed previously [20]. One important drawback of this method which is particularly significant to its applications for muscle modelling is that it is not possible to estimate optimal fiber lengths. This is often done in dissection studies using laser diffraction to measure sarcomere lengths [29], however this parameter is not directly measurable from DTI sequences. While optimal fiber lengths were calculated here based on previously published sarcomere lengths [3], combining DTI with further medical imaging such as micro-endoscopy [63, 64] to obtain *in vivo* sarcomere lengths from superficial muscles could provide more accurate estimates of optimal fiber length in future studies. Without a subject-specific correction for sarcomere length, the fiber length data presented here require further testing and optimization within the musculoskeletal models to ensure the muscles are operating at the correct part of the force-length curve [7].

It should be noted that measurement/observer error could have contributed to any lack of correlation seen in here between subject anthropometry and muscle architecture. This most likely had an effect during manual muscle segmentation to determine muscle volumes (and by extension calculate F_max_) and measuring pennation angles (which have little effect on musculoskeletal model output- see later discussion). However, as the determination of *in vivo* muscle fibre lengths was mostly automated and the same fiber tractography stopping criteria were used for each muscle of each subject, any errors in this parameter are likely due to variations in the quality of the diffusion tensor image images, rather than human error. As manual segmentation of vertebrate muscle is an often-used technique for measuring muscle volumes [6], and all the diffusion tensor images were pre-processed using the same method (see methods) before analysis, the effect of these potential errors on the overall results presented here was likely small.

The accuracy and limitations of the fiber tractography framework used here have been discussed previously [20]. However, these had a direct effect on the data presented here. While the average accuracy of the estimated muscle fiber lengths was <1 ± 7 mm, this was variable between subjects and between muscle groups (2 mm in the hip adductors, but 17 mm in the knee extensors). This variability could be due to diffusion tensor image quality or partial volume artefacts from bone or subcutaneous fat, which will have had variable affects depending on the location or size of the muscle being analyzed (see [45] for a detailed discussion on the possible sources of measurement variation in DTI fiber tractography). Despite this variation, even the larger average discrepancies in fiber lengths and pennation angles measurement accuracy mostly fall below the repeatability coefficients reported by Heemskerk et al. [45] (<50 mm for L_f_, <10.2° for θ), suggesting that this framework is relatively accurate and repeatable. However, it should be noted that the validation of this framework was performed on cadavers [20], which were not subject to the same physiological factors which may have affected the repeatability reported by Heemskerk et al. [45] (such as motion, breathing artefacts or body temperature), which could explain the relatively high accuracies previously reported. Nevertheless, while this method shows undoubted potential for the biomechanical modelling field, improving the consistency of the fiber tractography between muscles and individuals is needed for its widespread application.

Regarding pennation angles, this study reports lower angles than the limited three-dimensional angles derived from DTI tractography that are currently available in the literature. Values of ~30° have been previously reported in soleus and medial gastrocnemius muscles [53, 56], compared to mean values of 12° and 9° respectively reported here, which are more similar to angles measured from ultrasound or cadaveric data [3, 65–67]. While these differences to other DTI studies seem substantial, pennation angle is known to be highly dependent on joint position and has been estimated using DTI to change between 9° [56] and 46° [46] with 30° rotations in ankle dorsiflexion/plantarflexion. While the subjects in this study were asked to remain in the anatomical position during the image acquisition (with the hip, knee and ankle joints at 0° of flexion/extension), it is possible that the ankle joint was not exactly in this position for the duration of each scan. Even small deviations from a neutral position at the ankle joint could have caused large changes in pennation angles, particularly in the ankle dorsiflexor or plantarflexor muscles, and therefore could explain these differences. However, given the low sensitivity of muscle function predictions within musculoskeletal models to variations in the pennation angle input parameter [10, 13], the 2D surface pennation angles presented here could be sufficient in predicting muscle functions, if these data are to be used as inputs into such models. Nevertheless, further refinements to this framework for more accurately estimating optimal fiber lengths and pennation angles could be of benefit in future studies.

### Impact and future study

This study represents the first instance of an extensive data set of *in vivo* human lower limb muscle architecture generated purely from medical imaging (DTI and MRI), with a specific focus on implications for biomechanics and musculoskeletal modeling. By investigating the scaling relationships between anthropometric parameters and important muscle force generating properties such as muscle fiber length and maximum isometric force, these data show a lack of correlation between muscle fiber length and anthropometry amongst most muscle functional groups of the lower limb. This means that optimization or scaling algorithms often used to estimate muscle architecture for musculoskeletal modeling may not reliably do so, and that how muscle fiber lengths relate to body proportions may be more complex when compared to similar relationships with other muscle architecture variables. Nevertheless, given the differences between these data and previously published cadaveric architecture data, it is possible that applying the muscle parameters presented here to musculoskeletal models of individuals of similar age or anthropometry could provide more accurate estimates of muscle function than similar data from those previous studies. Furthermore, the accurate muscle fiber paths reconstructed using this method could also improve muscle functional predictions through more accurate representations of muscle moment arms, and can be incorporated into biomechanical models using methods such as that described by Chen et al., [68].

While this study focused on estimating muscle architecture for young, healthy individuals using DTI, this framework could further benefit the musculoskeletal modeling field by measuring similar parameters in pathological populations (e.g. individuals with cerebral palsy [54], muscle atrophy [69], muscular dystrophy [70], and the elderly [71]), whose gait and muscle function are often investigated with biomechanical models and simulations [72, 73].

Furthermore, the differences to previous data, as well as the variation within our data set, also lends support to the emergence of subject-specific musculoskeletal modeling. Although generic and scaled-generic models are generally effective at testing general predictions of musculoskeletal function, more detailed modeling analyses such as those predicting rehabilitation or post-surgical outcomes may require the inclusion of subject-specific muscle architecture data for maximum efficacy. Future work will focus on testing these assumptions and further validating this framework. Despite the methods used here to measure muscle architecture *in vivo* being previously validated specifically for use in musculoskeletal modelling [20], it is still unclear how accurately the data will simulate muscle functions within these models. The validity of these methods can be further assessed by comparing muscle forces predicted by subject-specific musculoskeletal models to those measured experimentally, such as from an isokinetic dynamometer. Accurate predictions of muscle forces from subject-specific models would further raise confidence in the validity of this framework in measuring muscle architecture *in vivo* and forming the basis of individualized musculoskeletal models.

## Supporting information

S1 Table Architecture properties of 20 lower limb muscles from Subject 01, plus functional group means (± standard deviations). Fiber lengths and pennation angles are expressed as means (± standard deviations) of multiple measurements taken at different areas of each muscle. L_f_:L_m_- muscle length fiber length ratio. PCSA- Physiological cross-sectional area. F_max_- estimated maximum isometric force. Sarcomere lengths used to estimate optimal fiber lengths were sourced from Ward et al., [3].
(DOCX)

S2 Table Architecture properties of 20 lower limb muscles from Subject 02, plus functional group means (± standard deviations). Fiber lengths and pennation angles are expressed as means (± standard deviations) of multiple measurements taken at different areas of each muscle. L_f_:L_m_- muscle length fiber length ratio. PCSA- Physiological cross-sectional area. F_max_- estimated maximum isometric force. Sarcomere lengths used to estimate optimal fiber lengths were sourced from Ward et al., [3].
(DOCX)

S3 Table Architecture properties of 20 lower limb muscles from Subject 03, plus functional group means (± standard deviations). Fiber lengths and pennation angles are expressed as means (± standard deviations) of multiple measurements taken at different areas of each muscle. L_f_:L_m_- muscle length fiber length ratio. PCSA- Physiological cross-sectional area. F_max_- estimated maximum isometric force. Sarcomere lengths used to estimate optimal fiber lengths were sourced from Ward et al., [3].
(DOCX)

S4 Table Architecture properties of 20 lower limb muscles from Subject 04, plus functional group means (± standard deviations). Fiber lengths and pennation angles are expressed as means (± standard deviations) of multiple measurements taken at different areas of each muscle. L_f_:L_m_- muscle length fiber length ratio. PCSA- Physiological cross-sectional area. F_max_- estimated maximum isometric force. Sarcomere lengths used to estimate optimal fiber lengths were sourced from Ward et al., [3].
(DOCX)

S5 Table Architecture properties of 20 lower limb muscles from Subject 05, plus functional group means (± standard deviations). Fiber lengths and pennation angles are expressed as means (± standard deviations) of multiple measurements taken at different areas of each muscle. L_f_:L_m_- muscle length fiber length ratio. PCSA- Physiological cross-sectional area. F_max_- estimated maximum isometric force. Sarcomere lengths used to estimate optimal fiber lengths were sourced from Ward et al., [3].
(DOCX)

S6 Table Architecture properties of 20 lower limb muscles from Subject 06, plus functional group means (± standard deviations). Fiber lengths and pennation angles are expressed as means (± standard deviations) of multiple measurements taken at different areas of each muscle. L_f_:L_m_- muscle length fiber length ratio. PCSA- Physiological cross-sectional area. F_max_- estimated maximum isometric force. Sarcomere lengths used to estimate optimal fiber lengths were sourced from Ward et al., [3].
(DOCX)

S7 Table Architecture properties of 20 lower limb muscles from Subject 07, plus functional group means (± standard deviations). Fiber lengths and pennation angles are expressed as means (± standard deviations) of multiple measurements taken at different areas of each muscle. L_f_:L_m_- muscle length fiber length ratio. PCSA- Physiological cross-sectional area. F_max_- estimated maximum isometric force. Sarcomere lengths used to estimate optimal fiber lengths were sourced from Ward et al., [3].
(DOCX)

S8 Table Architecture properties of 20 lower limb muscles from Subject 08, plus functional group means (± standard deviations). Fiber lengths and pennation angles are expressed as means (± standard deviations) of multiple measurements taken at different areas of each muscle. L_f_:L_m_- muscle length fiber length ratio. PCSA- Physiological cross-sectional area. F_max_- estimated maximum isometric force. Sarcomere lengths used to estimate optimal fiber lengths were sourced from Ward et al., [3].
(DOCX)

S9 Table Architecture properties of 20 lower limb muscles from Subject 09, plus functional group means (± standard deviations). Fiber lengths and pennation angles are expressed as means (± standard deviations) of multiple measurements taken at different areas of each muscle. L_f_:L_m_- muscle length fiber length ratio. PCSA- Physiological cross-sectional area. F_max_- estimated maximum isometric force. Sarcomere lengths used to estimate optimal fiber lengths were sourced from Ward et al., [3].
(DOCX)

S10 Table Architecture properties of 20 lower limb muscles from Subject 10, plus functional group means (± standard deviations). Fiber lengths and pennation angles are expressed as means (± standard deviations) of multiple measurements taken at different areas of each muscle. L_f_:L_m_- muscle length fiber length ratio. PCSA- Physiological cross-sectional area. F_max_- estimated maximum isometric force. Sarcomere lengths used to estimate optimal fiber lengths were sourced from Ward et al., [3].
(DOCX)

S11 Table Linear regression results (R^2^ values) to test the scaling relationships between muscle architecture parameters (L_f-_ muscle fiber length; L_f_:L_m_—muscle length fiber length ratio; F_max-_ estimated maximum isometric force; V_m-_ muscle volume; L_m_- muscle length) and subject age, height, body mass, total limb mass and lower limb length. P values are shown in parentheses. Values italicized and in bold indicate statistical significance (p ≤ 0.05).
(DOCX)

S12 Table Mean differences (% differences) between lower limb muscle architecture data derived here from MRI, and those from a previous cadaveric study [3]. Muscle masses from our data were estimated from muscle volumes. L_f_:L_m_- Muscle fiber length muscle length ratio. PCSA- Physiological cross-sectional area. F_max_- estimated maximum isometric force. Sarcomere lengths used to estimate optimal fiber lengths were sourced from Ward et al., [3].
(DOCX)

S1 Fig Mean L_f_:L_m_ (fiber length muscle length ratio) for each muscle functional group, for subjects 1–10. For muscle functional group classifications, see Table 1. Horizontal dashed line represents the mean value from Ward et al., [3].
(TIF)

S2 Fig Mean estimated maximum isometric force (F_max_; expressed as percent body weight) values for each muscle functional group, for subjects 1–10. For muscle functional group classifications, see Table 1. Horizontal dashed line represents the mean value from Ward et al., [3].
(TIF)

S1 File Comparison to previous architecture data. (DOCX)
