# Supplementary material for: In vivo human lower limb muscle architecture dataset obtained using diffusion tensor imaging
Source: PLoS One. 2019 Oct 15;14(10):e0223531. doi: 10.1371/journal.pone.0223531 (PMC6793854; doi:10.1371/journal.pone.0223531)
Supplement: S1 Table — Fiber lengths and pennation angles are expressed as means (± standard deviations) of multiple measurements taken at different areas of each muscle. Lf:Lm- muscle length fiber length ratio. PCSA- Physiological cross-sectional area. Fmax- estimated maximum isometric force. Sarcomere lengths used to estimate optimal fiber lengths were sourced from Ward et al., [3]. (DOCX) [file pone.0223531.s001.docx]

| **Muscle** | **Muscle Volume (cm^3^)** | **Belly Length (mm)** | **Optimal fiber length (mm)** | **L_f_:L_m_** | **Pennation angle (°)** | **PCSA (mm^2^)** | **F_max_ (N)** | **F_max_ (%BW)** |
| --- | --- | --- | --- | --- | --- | --- | --- | --- |
| **Adductor magnus** | 1007 | 300 | 228 ± 20 | 0.76 | 18 ± 4 | 4203 | 1261 | 142 |
| **Adductor longus** | 228 | 242 | 102 ± 40 | 0.42 | 13 ± 4 | 2172 | 652 | 73 |
| **Adductor brevis** | 122 | 163 | 61 ± 30 | 0.37 | 12 ± 3 | 1957 | 587 | 66 |
| **Gracilis** | 141 | 391 | 226 ± 34 | 0.58 | 7 ± 2 | 618 | 185 | 21 |
| **Semimembranosus** | 274 | 282 | 105 ± 85 | 0.37 | 20 ± 2 | 2444 | 733 | 82 |
| **Semitendinosus** | 272 | 355 | 169 ± 53 | 0.48 | 14 ± 2 | 1566 | 470 | 53 |
| **Biceps femoris- long head** | 323 | 273 | 128 ± 32 | 0.47 | 18 ± 4 | 2401 | 720 | 81 |
| **Biceps femoris- short head** | 134 | 307 | 107 ± 16 | 0.35 | 12 ± 4 | 1226 | 368 | 41 |
| **Popliteus** | 26 | 124 | 74 ± 20 | 0.59 | 19 ± 5 | 330 | 99 | 11 |
| **Sartorius** | 190 | 531 | 453 ± <1 | 0.85 | N/A | 420 | 126 | 14 |
| **Rectus femoris** | 322 | 329 | 111 ± 15 | 0.34 | 10 ± 2 | 2854 | 856 | 96 |
| **Vastus lateralis** | 900 | 357 | 115 ± 19 | 0.32 | 15 ± 3 | 7599 | 2280 | 256 |
| **Vastus medialis** | 638 | 367 | 119 ± 24 | 0.32 | 18 ± 10 | 5119 | 1536 | 173 |
| **Vastus intermedius** | 728 | 358 | 182 ± 53 | 0.51 | 11 ± 2 | 3923 | 1177 | 132 |
| **Tibialis anterior** | 146 | 380 | 167 ± 43 | 0.46 | 6 ± 1 | 867 | 260 | 29 |
| **Extensor digitorum longus** | 76 | 382 | 172 ± 47 | 0.47 | 7 ± 2 | 437 | 131 | 15 |
| **Extensor hallucis longus** | 29 | 247 | 105 ± 25 | 0.50 | 5 ± 2 | 276 | 83 | 9 |
| **Medial gastrocnemius** | 284 | 292 | 79 ± 17 | 0.27 | 11 ± 2 | 3327 | 998 | 112 |
| **Lateral gastrocnemius** | 141 | 329 | 143 ± 49 | 0.44 | 7 ± 4 | 970 | 291 | 33 |
| **Soleus** | 567 | 353 | 187 ± 9 | 0.56 | 13 ± 3 | 2947 | 884 | 99 |
| **Hip adductors** | **374 ± 367** | **274 ± 83** | **154 ± 74** | **0.53 ± 0.15** | **12 ± 4** | **2238 ± 1281** | **671 ± 384** | **75 ± 43** |
| **Knee flexors** | **239 ± 100** | **350 ± 121** | **173 ± 129** | **0.50 ± 0.17** | **14 ± 7** | **1398 ± 842** | **419 ± 253** | **47 ± 28** |
| **Knee extensors** | **647 ± 210** | **353 ± 14** | **132 ± 29** | **0.37 ± 0.08** | **14 ± 3** | **4874 ± 1766** | **1462 ± 530** | **164 ± 60** |
| **Ankle dorsiflexors** | **84 ± 48** | **336 ± 63** | **148 ± 31** | **0.48 ± 0.02** | **6 ± 1** | **527 ± 249** | **158 ± 75** | **18 ± 8** |
| **Ankle plantarflexors** | **331 ± 177** | **325 ± 25** | **139 ± 43** | **0.42 ± 0.12** | **11 ± 2** | **2414 ± 1033** | **724 ± 310** | **81 ± 35** |
