# Supplementary material for: In vivo human lower limb muscle architecture dataset obtained using diffusion tensor imaging
Source: PLoS One. 2019 Oct 15;14(10):e0223531. doi: 10.1371/journal.pone.0223531 (PMC6793854; doi:10.1371/journal.pone.0223531)
Supplement: S2 Table — Fiber lengths and pennation angles are expressed as means (± standard deviations) of multiple measurements taken at different areas of each muscle. Lf:Lm- muscle length fiber length ratio. PCSA- Physiological cross-sectional area. Fmax- estimated maximum isometric force. Sarcomere lengths used to estimate optimal fiber lengths were sourced from Ward et al., [3]. (DOCX) [file pone.0223531.s002.docx]

| **Muscle** |  | **Muscle Volume (cm^3^)** | **Belly Length (mm)** | **Optimal fiber length (mm)** | **L_f_:L_m_** | **Pennation angle (°)** | **PCSA (mm^2^)** | **F_max_ (N)** | **F_max_ (%BW)** |
| --- | --- | --- | --- | --- | --- | --- | --- | --- | --- |
| **Adductor magnus** |  | 654 | 274 | 311 ± 18 | 0.92 | 15 ± 3 | 2031 | 609 | 76 |
| **Adductor longus** |  | 163 | 188 | 125 ± 23 | 0.67 | 11 ± 2 | 1278 | 383 | 48 |
| **Adductor brevis** |  | 101 | 137 | 104 ± 23 | 0.75 | 15 ± 4 | 937 | 281 | 35 |
| **Gracilis** |  | 81 | 353 | 157 ± 74 | 0.44 | 7 ± 2 | 512 | 154 | 19 |
| **Semimembranosus** |  | 236 | 265 | 170 ± 45 | 0.64 | 10 ± 1 | 1368 | 410 | 51 |
| **Semitendinosus** |  | 194 | 331 | 99 ± 30 | 0.30 | 10 ± 2 | 1928 | 578 | 72 |
| **Biceps femoris- long head** |  | 196 | 250 | 190 ± 37 | 0.76 | 22 ± 4 | 954 | 286 | 36 |
| **Biceps femoris- short head** |  | 77 | 242 | 75 ± 10 | 0.31 | 9 ± 1 | 1012 | 304 | 38 |
| **Popliteus** |  | 16 | 87 | 58 ± 3 | 0.67 | 11 ± 5 | 269 | 81 | 10 |
| **Sartorius** |  | 133 | 533 | 400 ± <1 | 0.85 | N/A | 333 | 100 | 12 |
| **Rectus femoris** |  | 247 | 343 | 126 ± 35 | 0.37 | 10 ± 1 | 1933 | 580 | 72 |
| **Vastus lateralis** |  | 645 | 329 | 211 ± 52 | 0.64 | 27 ± 3 | 2724 | 817 | 101 |
| **Vastus medialis** |  | 503 | 326 | 103 ± 22 | 0.32 | 21 ± 4 | 4533 | 1360 | 169 |
| **Vastus intermedius** |  | 634 | 356 | 128 ± 20 | 0.36 | 21 ± 2 | 4624 | 1387 | 172 |
| **Tibialis anterior** |  | 149 | 373 | 134 ± 30 | 0.36 | 12 ± 4 | 1090 | 327 | 41 |
| **Extensor digitorum longus** |  | 93 | 351 | 103 ± 32 | 0.29 | 12 ± 2 | 880 | 264 | 33 |
| **Extensor hallucis longus** |  | 32 | 186 | 81 ± 30 | 0.43 | 10 ± 3 | 395 | 119 | 15 |
| **Medial gastrocnemius** |  | 237 | 257 | 88 ± 26 | 0.38 | 20 ± 4 | 2517 | 755 | 94 |
| **Lateral gastrocnemius** |  | 166 | 242 | 74 ± 44 | 0.33 | 16 ± 4 | 2158 | 647 | 80 |
| **Soleus** |  | 570 | 388 | 182 ± 19 | 0.47 | 10 ± 2 | 3092 | 927 | 115 |
| **Hip adductors** |  | **250 ± 236** | **238 ± 83** | **174 ± 81** | **0.70 ± 0.17** | **12 ± 3** | **1190 ± 556** | **357 ± 165** | **44 ± 21** |
| **Knee flexors** |  | **167 ± 76** | **324 ± 133** | **165 ± 115** | **0.57 ± 0.21** | **10 ± 6** | **977 ± 574** | **293 ± 180** | **36 ± 21** |
| **Knee extensors** |  | **507 ± 160** | **338 ± 12** | **142 ± 41** | **0.42 ± 0.13** | **20 ± 6** | **3453 ± 1160** | **1036 ± 310** | **129 ± 43** |
| **Ankle dorsiflexors** |  | **91 ± 48** | **303 ± 84** | **106 ± 22** | **0.36 ± 0.06** | **12 ± 1** | **788 ± 291** | **236 ± 87** | **29 ± 11** |
| **Ankle plantarflexors** |  | **325 ± 176** | **296 ± 66** | **115 ± 48** | **0.39 ± 0.06** | **16 ± 4** | **2589 ± 385** | **777 ± 135** | **96 ± 14** |
