# Supplementary material for: In vivo human lower limb muscle architecture dataset obtained using diffusion tensor imaging
Source: PLoS One. 2019 Oct 15;14(10):e0223531. doi: 10.1371/journal.pone.0223531 (PMC6793854; doi:10.1371/journal.pone.0223531)
Supplement: S3 Table — Fiber lengths and pennation angles are expressed as means (± standard deviations) of multiple measurements taken at different areas of each muscle. Lf:Lm- muscle length fiber length ratio. PCSA- Physiological cross-sectional area. Fmax- estimated maximum isometric force. Sarcomere lengths used to estimate optimal fiber lengths were sourced from Ward et al., [3]. (DOCX) [file pone.0223531.s003.docx]

| **Muscle** | **Muscle Volume (cm^3^)** | **Belly Length (mm)** | **Optimal fiber length (mm)** | **L_f_:L_m_** | **Pennation angle (°)** | **PCSA (mm^2^)** | **F_max_ (N)** | **Max force (%BW)** |
| --- | --- | --- | --- | --- | --- | --- | --- | --- |
| **Adductor magnus** | 533 | 321 | 271 ± 41 | 0.84 | 12 ± 1 | 1926 | 578 | 73 |
| **Adductor longus** | 206 | 262 | 105 ± 19 | 0.40 | 14 ± 2 | 1907 | 572 | 72 |
| **Adductor brevis** | 86 | 157 | 73 ± 27 | 0.47 | 9 ± 2 | 1167 | 350 | 44 |
| **Gracilis** | 100 | 334 | 212 ± 53 | 0.63 | N/A | 470 | 141 | 18 |
| **Semimembranosus** | 258 | 296 | 187 ± 62 | 0.63 | 13 ± 1 | 1343 | 403 | 51 |
| **Semitendinosus** | 258 | 324 | 158 ± 51 | 0.49 | 7 ± 2 | 1624 | 487 | 61 |
| **Biceps femoris- long head** | 241 | 269 | 213 ± 23 | 0.79 | 8 ± 3 | 1121 | 336 | 42 |
| **Biceps femoris- short head** | 109 | 285 | 158 ± 46 | 0.38 | 10 ± 3 | 996 | 299 | 38 |
| **Popliteus** | 19 | 124 | 95 ± 10 | 0.77 | 6 ± 2 | 198 | 59 | 7 |
| **Sartorius** | 150 | 551 | 434 ± <1 | 0.85 | N/A | 346 | 104 | 13 |
| **Rectus femoris** | 319 | 351 | 121 ± 25 | 0.35 | 8 ± 2 | 2602 | 781 | 98 |
| **Vastus lateralis** | 668 | 315 | 231 ± 30 | 0.67 | 13 ± 1 | 3058 | 918 | 115 |
| **Vastus medialis** | 411 | 238 | 177 ± 43 | 0.74 | 13 ± 5 | 2265 | 680 | 85 |
| **Vastus intermedius** | 597 | 332 | 144 ± 33 | 0.43 | 10 ± 1 | 4084 | 1225 | 154 |
| **Tibialis anterior** | 153 | 312 | 167 ± 22 | 0.54 | 7 ± 1 | 913 | 274 | 34 |
| **Extensor digitorum longus** | 72 | 323 | 127 ± 56 | 0.39 | 8 ± 1 | 559 | 168 | 21 |
| **Extensor hallucis longus** | 25 | 347 | 132 ± 56 | 0.38 | 8 ± 2 | 189 | 57 | 7 |
| **Medial gastrocnemius** | 232 | 234 | 105 ± 22 | 0.45 | 8 ± 1 | 2188 | 656 | 82 |
| **Lateral gastrocnemius** | 126 | 203 | 145 ± 47 | 0.72 | 9 ± 2 | 862 | 258 | 32 |
| **Soleus** | 474 | 353 | 108 ± 20 | 0.31 | 12 ± 2 | 4281 | 1284 | 161 |
| **Hip adductors** | **231 ± 181** | **268 ± 70** | **165 ± 80** | **0.59 ± 0.17** | **9 ± 5** | **1367 ± 602** | **410 ± 180** | **52 ± 23** |
| **Knee flexors** | **203 ± 89** | **345 ± 126** | **199 ± 113** | **0.63 ± 0.17** | **7 ± 4** | **938 ± 511** | **281 ± 153** | **35 ± 19** |
| **Knee extensors** | **499 ± 140** | **309 ± 43** | **164 ± 60** | **0.55 ± 0.16** | **11 ± 2** | **3002 ± 685** | **901 ± 205** | **113 ± 26** |
| **Ankle dorsiflexors** | **83 ± 53** | **327 ± 15** | **142 ± 36** | **0.44 ± 0.07** | **8 ± 0** | **554 ± 296** | **166 ± 89** | **21 ± 11** |
| **Ankle plantarflexors** | **278 ± 146** | **263 ± 65** | **120 ± 65** | **0.49 ± 0.17** | **10 ± 2** | **2443 ± 1407** | **733 ± 422** | **92 ± 53** |
