# Supplementary material for: In vivo human lower limb muscle architecture dataset obtained using diffusion tensor imaging
Source: PLoS One. 2019 Oct 15;14(10):e0223531. doi: 10.1371/journal.pone.0223531 (PMC6793854; doi:10.1371/journal.pone.0223531)
Supplement: S4 Table — Fiber lengths and pennation angles are expressed as means (± standard deviations) of multiple measurements taken at different areas of each muscle. Lf:Lm- muscle length fiber length ratio. PCSA- Physiological cross-sectional area. Fmax- estimated maximum isometric force. Sarcomere lengths used to estimate optimal fiber lengths were sourced from Ward et al., [3]. (DOCX) [file pone.0223531.s004.docx]

| **Muscle** | **Muscle Volume (cm^3^)** | **Belly Length (mm)** | **Optima fiber length (mm)** | **L_f_:L_m_** | **Pennation angle (°)** | **PCSA (mm^2^)** | **F_max_ (N)** | **F_max_ (%BW)** |
| --- | --- | --- | --- | --- | --- | --- | --- | --- |
| **Adductor magnus** | 549 | 274 | 146 ± 17 | 0.53 | 12 ± 3 | 3681 | 1104 | 158 |
| **Adductor longus** | 98 | 202 | 51 ± 14 | 0.25 | 11 ± 4 | 1891 | 567 | 81 |
| **Adductor brevis** | 64 | 123 | 34 ± 14 | 0.28 | 14 ± 3 | 1847 | 554 | 79 |
| **Gracilis** | 76 | 348 | 175 ± 49 | 0.50 | 6 ± 1 | 431 | 129 | 19 |
| **Semimembranosus** | 262 | 276 | 127 ± 14 | 0.46 | 9 ± 2 | 2037 | 611 | 87 |
| **Semitendinosus** | 154 | 355 | 228 ± 1 | 0.64 | 5 ± 1 | 673 | 202 | 29 |
| **Biceps femoris- long head** | 161 | 265 | 241 ± 25 | 0.91 | 10 ± 2 | 656 | 197 | 28 |
| **Biceps femoris- short head** | 91 | 271 | 137 ± 16 | 0.51 | 9 ± 1 | 656 | 197 | 28 |
| **Popliteus** | 12 | 62 | 55 ± 8 | 0.89 | 11 ± 3 | 209 | 63 | 9 |
| **Sartorius** | 169 | 458 | 389 ± <1 | 0.85 | N/A | 433 | 130 | 19 |
| **Rectus femoris** | 215 | 289 | 150 ± 27 | 0.52 | 7 ± 0 | 1419 | 426 | 61 |
| **Vastus lateralis** | 638 | 366 | 230 ± 26 | 0.63 | 15 ± 2 | 2679 | 804 | 115 |
| **Vastus medialis** | 404 | 325 | 210 ± 34 | 0.65 | 11 ± 2 | 1882 | 565 | 81 |
| **Vastus intermedius** | 470 | 343 | 215 ± 33 | 0.63 | 11 ± 2 | 2147 | 644 | 92 |
| **Tibialis anterior** | 95 | 259 | 109 ± 16 | 0.42 | 9 ± 2 | 862 | 259 | 37 |
| **Extensor digitorum longus** | 56 | 337 | 130 ± 47 | 0.39 | 7 ± 1 | 425 | 127 | 18 |
| **Extensor hallucis longus** | 20 | 249 | 128 ± 10 | 0.51 | 5 ± 1 | 158 | 47 | 7 |
| **Medial gastrocnemius** | 191 | 237 | 82 ± 34 | 0.35 | 11 ± 1 | 2293 | 688 | 98 |
| **Lateral gastrocnemius** | 128 | 203 | 74 ± 49 | 0.36 | 7 ± 2 | 1711 | 513 | 73 |
| **Soleus** | 358 | 292 | 118 ± 3 | 0.40 | 11 ± 3 | 2981 | 894 | 128 |
| **Hip adductors** | **197 ± 204** | **237 ± 84** | **101 ± 60** | **0.39 ± 0.13** | **11 ± 3** | **1962 ± 1153** | **589 ± 346** | **84 ± 50** |
| **Knee flexors** | **167 ± 77** | **325 ± 119** | **196 ± 107** | **0.67 ± 0.18** | **7 ± 4** | **777 ± 587** | **233 ± 176** | **33 ± 25** |
| **Knee extensors** | **432 ± 151** | **331 ± 28** | **201 ± 30** | **0.61 ± 0.05** | **11 ± 3** | **2032 ± 456** | **609 ± 137** | **87 ± 20** |
| **Ankle dorsiflexors** | **57 ± 30** | **282 ± 40** | **122 ± 10** | **0.44 ± 0.05** | **7 ± 1** | **482 ± 290** | **145 ± 87** | **21 ± 12** |
| **Ankle plantarflexors** | **226 ± 97** | **244 ± 37** | **91 ± 19** | **0.37 ± 0.02** | **10 ± 2** | **2328 ± 519** | **699 ± 156** | **100 ± 22** |
