# Supplementary material for: In vivo human lower limb muscle architecture dataset obtained using diffusion tensor imaging
Source: PLoS One. 2019 Oct 15;14(10):e0223531. doi: 10.1371/journal.pone.0223531 (PMC6793854; doi:10.1371/journal.pone.0223531)
Supplement: S5 Table — Fiber lengths and pennation angles are expressed as means (± standard deviations) of multiple measurements taken at different areas of each muscle. Lf:Lm- muscle length fiber length ratio. PCSA- Physiological cross-sectional area. Fmax- estimated maximum isometric force. Sarcomere lengths used to estimate optimal fiber lengths were sourced from Ward et al., [3]. (DOCX) [file pone.0223531.s005.docx]

| **Muscle** | **Muscle Volume (cm^3^)** | **Belly Length (mm)** | **Optimal fiber length (mm)** | **L_f_:L_m_** | **Pennation angle (°)** | **PCSA (mm^2^)** | **F_max_ (N)** | **F_max_ (%BW)** |
| --- | --- | --- | --- | --- | --- | --- | --- | --- |
| **Adductor magnus** | 381 | 258 | 177 ± 33 | 0.68 | 14 ± 4 | 2097 | 629 | 107 |
| **Adductor longus** | 147 | 231 | 145 ± 37 | 0.63 | 13 ± 3 | 987 | 296 | 50 |
| **Adductor brevis** | 100 | 160 | 100 ± 27 | 0.62 | 10 ± 3 | 983 | 295 | 50 |
| **Gracilis** | 82 | 344 | 109 ± 27 | 0.32 | 6 ± 2 | 748 | 224 | 38 |
| **Semimembranosus** | 214 | 257 | 176 ± 67 | 0.68 | 10 ± 1 | 1198 | 359 | 61 |
| **Semitendinosus** | 178 | 309 | 237 ± 33 | 0.77 | 7 ± 1 | 746 | 224 | 38 |
| **Biceps femoris- long head** | 154 | 234 | 198 ± 36 | 0.97 | 9 ± 2 | 666 | 200 | 34 |
| **Biceps femoris- short head** | 77 | 274 | 107 ± 41 | 0.39 | 8 ± 2 | 710 | 213 | 36 |
| **Popliteus** | 11 | 95 | 94 ± 12 | 0.99 | 9 ± 2 | 115 | 34 | 6 |
| **Sartorius** | 113 | 509 | 394 ± <1 | 0.85 | N/A | 287 | 86 | 15 |
| **Rectus femoris** | 273 | 315 | 140 ± 16 | 0.44 | 8 ± 2 | 1934 | 580 | 99 |
| **Vastus lateralis** | 553 | 319 | 152 ± 28 | 0.47 | 16 ± 4 | 3514 | 1054 | 179 |
| **Vastus medialis** | 332 | 326 | 146 ± 20 | 0.45 | 13 ± 2 | 2218 | 665 | 113 |
| **Vastus intermedius** | 474 | 353 | 165 ± 10 | 0.47 | 14 ± 2 | 2793 | 838 | 143 |
| **Tibialis anterior** | 130 | 299 | 100 ± 35 | 0.33 | 7 ± 2 | 1287 | 386 | 66 |
| **Extensor digitorum longus** | 88 | 357 | 101 ± 18 | 0.28 | 5 ± 1 | 866 | 260 | 44 |
| **Extensor hallucis longus** | 5 | 176 | 68 ± 40 | 0.38 | 4 ± 1 | 76 | 23 | 4 |
| **Medial gastrocnemius** | 202 | 270 | 89 ± 42 | 0.33 | 9 ± 1 | 2244 | 673 | 115 |
| **Lateral gastrocnemius** | 101 | 239 | 128 ± 43 | 0.54 | 7 ± 1 | 781 | 234 | 40 |
| **Soleus** | 375 | 355 | 140 ± 17 | 0.39 | 12 ± 2 | 2626 | 788 | 134 |
| **Hip adductors** | **177 ± 120** | **248 ± 66** | **133 ± 30** | **0.56 ± 0.14** | **11 ± 3** | **1204 ± 525** | **361 ± 157** | **61 ± 27** |
| **Knee flexors** | **147 ± 67** | **316 ± 123** | **206 ± 100** | **0.73 ± 0.20** | **7 ± 3** | **620 ± 348** | **186 ± 104** | **32 ± 18** |
| **Knee extensors** | **408 ± 111** | **328 ± 15** | **150 ± 9** | **0.46 ± 0.01** | **13 ± 3** | **2614 ± 605** | **784 ± 181** | **134 ± 31** |
| **Ankle dorsiflexors** | **74 ± 52** | **277 ± 76** | **90 ± 16** | **0.33 ± 0.04** | **5 ± 1** | **743 ± 502** | **223 ± 151** | **38 ± 26** |
| **Ankle plantarflexors** | **226 ± 113** | **288 ± 49** | **119 ± 12** | **0.42 ± 0.09** | **9 ± 2** | **1883 ± 795** | **565 ± 239** | **96 ± 41** |
