# Supplementary material for: In vivo human lower limb muscle architecture dataset obtained using diffusion tensor imaging
Source: PLoS One. 2019 Oct 15;14(10):e0223531. doi: 10.1371/journal.pone.0223531 (PMC6793854; doi:10.1371/journal.pone.0223531)
Supplement: S6 Table — Fiber lengths and pennation angles are expressed as means (± standard deviations) of multiple measurements taken at different areas of each muscle. Lf:Lm- muscle length fiber length ratio. PCSA- Physiological cross-sectional area. Fmax- estimated maximum isometric force. Sarcomere lengths used to estimate optimal fiber lengths were sourced from Ward et al., [3]. (DOCX) [file pone.0223531.s006.docx]

| **Muscle** | **Muscle Volume (cm^3^)** | **Belly Length (mm)** | **Optimal fiber length (mm)** | **L_f_:L_m_** | **Pennation angle (°)** | **PCSA (mm^2^)** | **F_max_ (N)** | **F_max_ (%BW)** |
| --- | --- | --- | --- | --- | --- | --- | --- | --- |
| **Adductor magnus** | 582 | 333 | 250 ± 51 | 0.75 | 11 ± 1 | 2286 | 686 | 87 |
| **Adductor longus** | 122 | 211 | 136 ± 11 | 0.64 | 10 ± 2 | 882 | 265 | 34 |
| **Adductor brevis** | 125 | 223 | 77 ± 20 | 0.35 | 11 ± 2 | 1590 | 477 | 61 |
| **Gracilis** | 87 | 349 | 156 ± 24 | 0.45 | 8 ± 1 | 550 | 165 | 21 |
| **Semimembranosus** | 269 | 235 | 193 ± 68 | 0.82 | 10 ± 2 | 1374 | 412 | 52 |
| **Semitendinosus** | 141 | 275 | 213 ± 28 | 0.77 | 8 ± 1 | 658 | 197 | 25 |
| **Biceps femoris- long head** | 188 | 280 | 210 ± 41 | 0.75 | 8 ± 1 | 888 | 266 | 34 |
| **Biceps femoris- short head** | 68 | 240 | 118 ± 12 | 0.49 | 7 ± 2 | 572 | 172 | 22 |
| **Popliteus** | 15 | 126 | 60 ± 12 | 0.48 | 9 ± 2 | 253 | 76 | 10 |
| **Sartorius** | 136 | 444 | 378 ± <1 | 0.85 | N/A | 359 | 108 | 14 |
| **Rectus femoris** | 203 | 308 | 218 ± 12 | 0.71 | 8 ± 5 | 924 | 277 | 35 |
| **Vastus lateralis** | 532 | 308 | 274 ± 10 | 0.89 | 16 ± 1 | 1865 | 559 | 71 |
| **Vastus medialis** | 375 | 325 | 147 ± 30 | 0.45 | 15 ± 4 | 2463 | 739 | 94 |
| **Vastus intermedius** | 522 | 358 | 228 ± 25 | 0.64 | 10 ± 2 | 2261 | 678 | 86 |
| **Tibialis anterior** | 127 | 267 | 101 ± 30 | 0.38 | 8 ± 2 | 1251 | 375 | 48 |
| **Extensor digitorum longus** | 70 | 320 | 136 ± 61 | 0.42 | 8 ± 2 | 511 | 153 | 19 |
| **Extensor hallucis longus** | 18 | 254 | 97 ± 53 | 0.38 | 8 ± 2 | 185 | 55 | 7 |
| **Medial gastrocnemius** | 249 | 239 | 121 ± 10 | 0.51 | 7 ± 1 | 2043 | 613 | 78 |
| **Lateral gastrocnemius** | 121 | 302 | 159 ± 48 | 0.53 | 7 ± 1 | 754 | 226 | 29 |
| **Soleus** | 435 | 310 | 157 ± 30 | 0.51 | 11 ± 2 | 2718 | 815 | 104 |
| **Hip adductors** | **229 ± 204** | **279 ± 62** | **155 ± 62** | **0.55 ± 0.16** | **10 ± 1** | **1327 ± 669** | **398 ± 201** | **51 ± 26** |
| **Knee flexors** | **160 ± 81** | **295 ± 94** | **195 ± 98** | **0.74 ± 0.15** | **7 ± 3** | **684 ± 370** | **205 ± 111** | **26 ± 14** |
| **Knee extensors** | **408 ± 134** | **325 ± 20** | **217 ± 45** | **0.67 ± 0.16** | **12 ± 4** | **1878 ± 591** | **564 ± 177** | **72 ± 23** |
| **Ankle dorsiflexors** | **72 ± 44** | **280 ± 29** | **111 ± 18** | **0.39 ± 0.02** | **8 ± 0** | **649 ± 446** | **195 ± 134** | **25 ± 17** |
| **Ankle plantarflexors** | **269 ± 129** | **284 ± 32** | **146 ± 18** | **0.51 ± 0.01** | **8 ± 2** | **1838 ± 815** | **551 ± 244** | **70 ± 31** |
