# Supplementary material for: In vivo human lower limb muscle architecture dataset obtained using diffusion tensor imaging
Source: PLoS One. 2019 Oct 15;14(10):e0223531. doi: 10.1371/journal.pone.0223531 (PMC6793854; doi:10.1371/journal.pone.0223531)
Supplement: S7 Table — Fiber lengths and pennation angles are expressed as means (± standard deviations) of multiple measurements taken at different areas of each muscle. Lf:Lm- muscle length fiber length ratio. PCSA- Physiological cross-sectional area. Fmax- estimated maximum isometric force. Sarcomere lengths used to estimate optimal fiber lengths were sourced from Ward et al., [3]. (DOCX) [file pone.0223531.s007.docx]

| **Muscle** | **Muscle Volume (cm^3^)** | **Belly Length (mm)** | **Optimal fiber length (mm)** | **L_f_:L_m_** | **Pennation angle (°)** | **PCSA (mm^2^)** | **F_max_ (N)** | **F_max_ (%BW)** |
| --- | --- | --- | --- | --- | --- | --- | --- | --- |
| **Adductor magnus** | 333 | 340 | 315 ± 52 | 0.90 | 9 ± 2 | 1072 | 322 | 41 |
| **Adductor longus** | 89 | 177 | 140 ± 29 | 0.71 | 9 ± 2 | 696 | 209 | 26 |
| **Adductor brevis** | 66 | 135 | 82 ± 22 | 0.53 | 12 ± 5 | 905 | 272 | 34 |
| **Gracilis** | 60 | 279 | 131 ± 12 | 0.47 | 8 ± 1 | 456 | 137 | 17 |
| **Semimembranosus** | 160 | 229 | 111 ± 13 | 0.47 | 12 ± 2 | 1456 | 437 | 55 |
| **Semitendinosus** | 121 | 333 | 151 ± 12 | 0.47 | 7 ± 1 | 772 | 232 | 29 |
| **Biceps femoris- long head** | 142 | 209 | 145 ± 4 | 0.69 | 11 ± 4 | 965 | 290 | 37 |
| **Biceps femoris- short head** | 91 | 209 | 184 ± 45 | 0.72 | 9 ± 2 | 598 | 179 | 23 |
| **Popliteus** | 10 | 97 | 87 ± 6 | 0.89 | 12 ± 4 | 114 | 34 | 4 |
| **Sartorius** | 129 | 411 | 368 ± <1 | 0.85 | N/A | 370 | 111 | 14 |
| **Rectus femoris** | 153 | 287 | 149 ± 35 | 0.46 | 5 ± 2 | 1157 | 347 | 44 |
| **Vastus lateralis** | 413 | 316 | 169 ± 20 | 0.54 | 10 ± 2 | 2377 | 713 | 90 |
| **Vastus medialis** | 250 | 347 | 208 ± 45 | 0.54 | 9 ± 3 | 1307 | 392 | 49 |
| **Vastus intermedius** | 297 | 309 | 214 ± 9 | 0.63 | 7 ± 3 | 1514 | 454 | 57 |
| **Tibialis anterior** | 117 | 242 | 196 ± 10 | 0.55 | 5 ± 1 | 865 | 260 | 33 |
| **Extensor digitorum longus** | 50 | 311 | 153 ± 24 | 0.49 | 7 ± 1 | 322 | 97 | 12 |
| **Extensor hallucis longus** | 21 | 262 | 161 ± 37 | 0.44 | 6 ± 2 | 181 | 54 | 7 |
| **Medial gastrocnemius** | 217 | 229 | 132 ± 21 | 0.36 | 8 ± 1 | 2591 | 777 | 98 |
| **Lateral gastrocnemius** | 93 | 184 | 84 ± 28 | 0.29 | 9 ± 2 | 1720 | 516 | 65 |
| **Soleus** | 341 | 346 | 223 ± 9 | 0.49 | 8 ± 2 | 1983 | 595 | 75 |
| **Hip adductors** | **137 ± 114** | **233 ± 81** | **167 ± 88** | **0.65 ± 0.17** | **9 ± 2** | **782 ± 231** | **235 ± 69** | **30 ± 9** |
| **Knee flexors** | **129 ± 49** | **278 ± 100** | **180 ± 94** | **0.64 ± 0.17** | **8 ± 4** | **712 ± 430** | **214 ± 129** | **27 ± 16** |
| **Knee extensors** | **278 ± 94** | **315 ± 22** | **185 ± 27** | **0.54 ± 0.06** | **8 ± 2** | **1588 ± 472** | **477 ± 142** | **60 ± 18** |
| **Ankle dorsiflexors** | **62 ± 40** | **272 ± 29** | **170 ± 19** | **0.50 ± 0.05** | **6 ± 1** | **456 ± 295** | **137 ± 88** | **17 ± 11** |
| **Ankle plantarflexors** | **217 ± 101** | **253 ± 68** | **146 ± 58** | **0.38 ± 0.08** | **8 ± 0** | **2098 ± 365** | **629 ± 109** | **79 ± 14** |
