# Supplementary material for: In vivo human lower limb muscle architecture dataset obtained using diffusion tensor imaging
Source: PLoS One. 2019 Oct 15;14(10):e0223531. doi: 10.1371/journal.pone.0223531 (PMC6793854; doi:10.1371/journal.pone.0223531)
Supplement: S8 Table — Fiber lengths and pennation angles are expressed as means (± standard deviations) of multiple measurements taken at different areas of each muscle. Lf:Lm- muscle length fiber length ratio. PCSA- Physiological cross-sectional area. Fmax- estimated maximum isometric force. Sarcomere lengths used to estimate optimal fiber lengths were sourced from Ward et al., [3]. (DOCX) [file pone.0223531.s008.docx]

| **Muscle** | **Muscle Volume (cm^3^)** | **Belly Length (mm)** | **Optimal fiber length (mm)** | **L_f_:L_m_** | **Pennation angle (°)** | **PCSA (mm^2^)** | **F_max_ (N)** | **F_max_ (%BW)** |
| --- | --- | --- | --- | --- | --- | --- | --- | --- |
| **Adductor magnus** | 366 | 315 | 120 ± 7 | 0.38 | 10 ± 2 | 3006 | 902 | 197 |
| **Adductor longus** | 100 | 197 | 77 ± 20 | 0.39 | 10 ± 4 | 1281 | 384 | 84 |
| **Adductor brevis** | 66 | 125 | 53 ± 12 | 0.42 | 9 ± 2 | 1232 | 369 | 81 |
| **Gracilis** | 43 | 316 | 74 ± 15 | 0.23 | 8 ± 1 | 576 | 173 | 38 |
| **Semimembranosus** | 155 | 247 | 114 ± 32 | 0.46 | 15 ± 3 | 1311 | 393 | 86 |
| **Semitendinosus** | 110 | 288 | 134 ± 25 | 0.47 | 8 ± 3 | 815 | 244 | 53 |
| **Biceps femoris- long head** | 117 | 285 | 237 ± 25 | 0.83 | 10 ± 1 | 485 | 146 | 32 |
| **Biceps femoris- short head** | 69 | 308 | 82 ± 25 | 0.27 | 9 ± 3 | 826 | 248 | 54 |
| **Popliteus** | 8 | 78 | 73 ± 9 | 0.93 | 8 ± 1 | 95 | 28 | 6 |
| **Sartorius** | 69 | 503 | 407 ± <1 | 0.85 | N/A | 168 | 51 | 11 |
| **Rectus femoris** | 154 | 290 | 63 ± 6 | 0.22 | 8 ± 1 | 2402 | 721 | 157 |
| **Vastus lateralis** | 393 | 323 | 187 ± 41 | 0.58 | 13 ± 4 | 2045 | 614 | 134 |
| **Vastus medialis** | 277 | 313 | 114 ± 26 | 0.37 | 12 ± 4 | 2379 | 714 | 156 |
| **Vastus intermedius** | 343 | 337 | 115 ± 14 | 0.34 | 11 ± 2 | 2944 | 883 | 193 |
| **Tibialis anterior** | 86 | 272 | 140 ± 16 | 0.52 | 6 ± 1 | 607 | 182 | 40 |
| **Extensor digitorum longus** | 61 | 320 | 143 ± 42 | 0.45 | 7 ± 2 | 426 | 128 | 28 |
| **Extensor hallucis longus** | 15 | 209 | 79 ± 42 | 0.38 | 7 ± 2 | 191 | 57 | 13 |
| **Medial gastrocnemius** | 129 | 225 | 69 ± 16 | 0.31 | 11 ± 2 | 1836 | 551 | 120 |
| **Lateral gastrocnemius** | 69 | 196 | 88 ± 12 | 0.45 | 12 ± 2 | 765 | 230 | 50 |
| **Soleus** | 325 | 350 | 149 ± 11 | 0.43 | 16 ± 4 | 2100 | 630 | 137 |
| **Hip adductors** | **144 ± 130** | **238 ± 81** | **81 ± 24** | **0.36 ± 0.07** | **9 ± 1** | **1524 ± 900** | **457 ± 270** | **100 ± 59** |
| **Knee flexors** | **104 ± 47** | **326 ± 124** | **176 ± 116** | **0.58 ± 0.26** | **8 ± 4** | **617 ± 403** | **185 ± 126** | **40 ± 27** |
| **Knee extensors** | **292 ± 90** | **316 ± 17** | **120 ± 44** | **0.38 ± 0.13** | **11 ± 2** | **2443 ± 241** | **733 ± 97** | **160 ± 21** |
| **Ankle dorsiflexors** | **54 ± 29** | **267 ± 45** | **121 ± 30** | **0.45 ± 0.06** | **7 ± 1** | **408 ± 210** | **122 ± 51** | **27 ± 11** |
| **Ankle plantarflexors** | **174 ± 109** | **257 ± 67** | **102 ± 34** | **0.39 ± 0.06** | **13 ± 2** | **1567 ± 655** | **470 ± 173** | **103 ± 38** |
