# Supplementary material for: In vivo human lower limb muscle architecture dataset obtained using diffusion tensor imaging
Source: PLoS One. 2019 Oct 15;14(10):e0223531. doi: 10.1371/journal.pone.0223531 (PMC6793854; doi:10.1371/journal.pone.0223531)
Supplement: S9 Table — Fiber lengths and pennation angles are expressed as means (± standard deviations) of multiple measurements taken at different areas of each muscle. Lf:Lm- muscle length fiber length ratio. PCSA- Physiological cross-sectional area. Fmax- estimated maximum isometric force. Sarcomere lengths used to estimate optimal fiber lengths were sourced from Ward et al., [3]. (DOCX) [file pone.0223531.s009.docx]

| **Muscle** | **Muscle Volume (cm^3^)** | **Belly Length (mm)** | **Optimal fiber length (mm)** | **L_f_:L_m_** | **Pennation angle (°)** | **PCSA (mm^2^)** | **F_max_ (N)** | **F_max_ (%BW)** |
| --- | --- | --- | --- | --- | --- | --- | --- | --- |
| **Adductor magnus** | 689 | 270 | 262 ± 3 | 0.97 | 9 ± 1 | 2603 | 781 | 94 |
| **Adductor longus** | 264 | 249 | 112 ± 15 | 0.45 | 14 ± 5 | 2281 | 684 | 82 |
| **Adductor brevis** | 103 | 160 | 99 ± 23 | 0.62 | 9 ± 3 | 1033 | 310 | 37 |
| **Gracilis** | 156 | 358 | 263 ± 56 | 0.73 | 7 ± 2 | 588 | 176 | 21 |
| **Semimembranosus** | 363 | 319 | 247 ± 35 | 0.78 | 11 ± 1 | 1440 | 432 | 52 |
| **Semitendinosus** | 252 | 353 | 233 ± 36 | 0.66 | 7 ± 1 | 1078 | 323 | 39 |
| **Biceps femoris- long head** | 203 | 239 | 213 ± 40 | 0.89 | 9 ± 3 | 815 | 244 | 29 |
| **Biceps femoris- short head** | 126 | 351 | 109 ± 23 | 0.31 | 10 ± 2 | 1142 | 343 | 41 |
| **Popliteus** | 21 | 113 | 75 ± 19 | 0.66 | 9 ± 1 | 273 | 82 | 10 |
| **Sartorius** | 212 | 567 | 434 ± <1 | 0.85 | N/A | 489 | 147 | 18 |
| **Rectus femoris** | 350 | 369 | 209 ± 27 | 0.57 | 9 ± 3 | 1656 | 497 | 60 |
| **Vastus lateralis** | 795 | 365 | 214 ± 27 | 0.59 | 14 ± 3 | 3609 | 1083 | 130 |
| **Vastus medialis** | 556 | 390 | 224 ± 84 | 0.57 | 14 ± 5 | 2412 | 724 | 87 |
| **Vastus intermedius** | 585 | 362 | 227 ± 36 | 0.63 | 9 ± 2 | 2540 | 762 | 92 |
| **Tibialis anterior** | 151 | 295 | 149 ± 29 | 0.50 | 5 ± 1 | 1012 | 304 | 36 |
| **Extensor digitorum longus** | 102 | 393 | 183 ± 69 | 0.46 | 5 ± 1 | 555 | 167 | 20 |
| **Extensor hallucis longus** | 20 | 214 | 143 ± 77 | 0.67 | 5 ± 1 | 138 | 41 | 5 |
| **Medial gastrocnemius** | 311 | 296 | 145 ± 21 | 0.49 | 7 ± 2 | 2122 | 637 | 77 |
| **Lateral gastrocnemius** | 197 | 266 | 188 ± 43 | 0.71 | 9 ± 3 | 1038 | 311 | 37 |
| **Soleus** | 664 | 370 | 155 ± 20 | 0.42 | 14 ± 3 | 4147 | 1244 | 150 |
| **Hip adductors** | **303 ± 230** | **259 ± 71** | **184 ± 78** | **0.69 ± 0.19** | **10 ± 3** | **1626 ± 839** | **488 ± 252** | **59 ± 30** |
| **Knee flexors** | **231 ± 106** | **366 ± 137** | **224 ± 116** | **0.70 ± 0.19** | **8 ± 4** | **873 ± 397** | **262 ± 119** | **31 ± 14** |
| **Knee extensors** | **572 ± 158** | **371 ± 11** | **219 ± 7** | **0.59 ± 0.02** | **11 ± 3** | **2554 ± 697** | **766 ± 209** | **92 ± 25** |
| **Ankle dorsiflexors** | **91 ± 54** | **301 ± 73** | **158 ± 18** | **0.55 ± 0.09** | **5 ± 0** | **568 ± 357** | **170 ± 107** | **20 ± 13** |
| **Ankle plantarflexors** | **391 ± 199** | **310 ± 44** | **163 ± 18** | **0.54 ± 0.12** | **10 ± 3** | **2436 ± 1289** | **731 ± 387** | **88 ± 46** |
