# Supplementary material for: In vivo human lower limb muscle architecture dataset obtained using diffusion tensor imaging
Source: PLoS One. 2019 Oct 15;14(10):e0223531. doi: 10.1371/journal.pone.0223531 (PMC6793854; doi:10.1371/journal.pone.0223531)
Supplement: S10 Table — Fiber lengths and pennation angles are expressed as means (± standard deviations) of multiple measurements taken at different areas of each muscle. Lf:Lm- muscle length fiber length ratio. PCSA- Physiological cross-sectional area. Fmax- estimated maximum isometric force. Sarcomere lengths used to estimate optimal fiber lengths were sourced from Ward et al., [3]. (DOCX) [file pone.0223531.s010.docx]

| **Muscle** | **Muscle Volume (cm^3^)** | **Belly Length (mm)** | **Optimal fiber length (mm)** | **L_f_:L_m_** | **Pennation angle (°)** | **PCSA (mm^2^)** | **F_max_ (N)** | **F_max_ (%BW)** |
| --- | --- | --- | --- | --- | --- | --- | --- | --- |
| **Adductor magnus** | 572 | 345 | 239 ± 27 | 0.69 | 12 ± 2 | 2337 | 701 | 87 |
| **Adductor longus** | 170 | 228 | 124 ± 28 | 0.55 | 14 ± 1 | 1324 | 397 | 49 |
| **Adductor brevis** | 95 | 130 | 90 ± 19 | 0.70 | 14 ± 5 | 1024 | 307 | 38 |
| **Gracilis** | 82 | 362 | 223 ± 61 | 0.62 | 8 ± 3 | 364 | 109 | 14 |
| **Semimembranosus** | 253 | 312 | 152 ± 44 | 0.49 | 10 ± 1 | 1643 | 493 | 61 |
| **Semitendinosus** | 183 | 322 | 209 ± 55 | 0.65 | 6 ± 1 | 869 | 261 | 32 |
| **Biceps femoris- long head** | 216 | 303 | 207 ± 53 | 0.68 | 9 ± 2 | 1029 | 309 | 38 |
| **Biceps femoris- short head** | 76 | 303 | 99 ± 20 | 0.33 | 11 ± 3 | 751 | 225 | 28 |
| **Popliteus** | 10 | 71 | 65 ± 18 | 0.91 | 8 ± 1 | 160 | 48 | 6 |
| **Sartorius** | 125 | 530 | 436 ± <1 | 0.85 | N/A | 287 | 86 | 11 |
| **Rectus femoris** | 255 | 347 | 153 ± 28 | 0.44 | 8 ± 3 | 1650 | 495 | 61 |
| **Vastus lateralis** | 522 | 354 | 196 ± 39 | 0.56 | 13 ± 3 | 2587 | 776 | 96 |
| **Vastus medialis** | 406 | 405 | 158 ± 21 | 0.39 | 15 ± 4 | 2492 | 748 | 92 |
| **Vastus intermedius** | 559 | 424 | 215 ± 20 | 0.51 | 11 ± 2 | 2551 | 765 | 95 |
| **Tibialis anterior** | 136 | 303 | 163 ± 50 | 0.54 | 5 ± 1 | 831 | 249 | 31 |
| **Extensor digitorum longus** | 94 | 389 | 126 ± 61 | 0.32 | 7 ± 1 | 742 | 223 | 28 |
| **Extensor hallucis longus** | 21 | 241 | 97 ± 52 | 0.40 | 7 ± 2 | 211 | 63 | 8 |
| **Medial gastrocnemius** | 248 | 257 | 103 ± 42 | 0.40 | 8 ± 3 | 2372 | 712 | 88 |
| **Lateral gastrocnemius** | 138 | 240 | 166 ± 14 | 0.69 | 7 ± 1 | 821 | 246 | 30 |
| **Soleus** | 497 | 379 | 89 ± 20 | 0.23 | 10 ± 2 | 5511 | 1653 | 204 |
| **Hip adductors** | **230 ± 200** | **266 ± 94** | **169 ± 63** | **0.64 ± 0.06** | **12 ± 3** | **1262 ± 711** | **379 ± 213** | **47 ± 26** |
| **Knee flexors** | **170 ± 83** | **354 ± 133** | **195 ± 120** | **0.60 ± 0.20** | **7 ± 4** | **790 ± 490** | **237 ± 147** | **29 ± 18** |
| **Knee extensors** | **435 ± 119** | **382 ± 33** | **180 ± 26** | **0.47 ± 0.06** | **12 ± 2** | **2320 ± 388** | **696 ± 116** | **86 ± 14** |
| **Ankle dorsiflexors** | **83 ± 48** | **311 ± 61** | **128 ± 27** | **0.42 ± 0.09** | **6 ± 1** | **595 ± 274** | **178 ± 82** | **22 ± 10** |
| **Ankle plantarflexors** | **294 ± 150** | **292 ± 62** | **119 ± 34** | **0.44 ± 0.19** | **9 ± 1** | **2901 ± 1951** | **870 ± 585** | **108 ± 72** |
