# Supplementary material for: In vivo human lower limb muscle architecture dataset obtained using diffusion tensor imaging
Source: PLoS One. 2019 Oct 15;14(10):e0223531. doi: 10.1371/journal.pone.0223531 (PMC6793854; doi:10.1371/journal.pone.0223531)
Supplement: S11 Table — P values are shown in parentheses. Values italicized and in bold indicate statistical significance (p ≤ 0.05). (DOCX) [file pone.0223531.s011.docx]

| **R^2^ values** | **Subject Age** | **Subject height** | **Body mass** | **Total limb volume** | **Limb length** |
| --- | --- | --- | --- | --- | --- |
| **Hip adductors** |  |  |  |  |  |
| **L_f_** | 0.06 (0.49) | ***0.56 (0.01)*** | ***0.70 (<0.01)*** | ***0.40 (0.04)*** | ***0.49 (0.02)*** |
| **L_f_:L_m_** | 0.06 (0.49) | 0.28 (0.11) | 0.37 (0.06) | 0.10 (0.36) | 0.17 (0.23) |
| **F_max_** | 0.06 (0.49) | 0.01 (0.81) | 0.01 (0.10) | 0.32 (0.08) | 0.07 (0.47) |
| **V_m_** | 0.00 (0.87) | ***0.40 (0.04)*** | 0.49 (0.02) | ***0.94 (<0.01)*** | ***0.51 (0.02)*** |
| **L_m_** | 0.28 (0.11) | 0.38 (0.05) | 0.24 (0.14) | 0.34 (0.07) | 0.26 (0.13) |
| **Knee flexors** |  |  |  |  |  |
| **L_f_** | 0.07 (0.45) | 0.12 (0.32) | 0.00 (0.96) | 0.07 (0.45) | 0.25 (0.14) |
| **L_f_:L_m_** | 0.01 (0.80) | 0.04 (0.57) | 0.33 (0.08) | 0.23 (0.16) | 0.02 (0.69) |
| **F_max_** | 0.07 (0.44) | 0.24 (0.15) | ***0.45 (0.03)*** | ***0.68 (<0.01)*** | 0.27 (0.12) |
| **V_m_** | 0.00 (0.85) | ***0.49 (0.02)*** | ***0.55 (0.01)*** | ***0.93 (<0.01)*** | ***0.66 (<0.01)*** |
| **L_m_** | 0.02 (0.66) | 0.13 (0.30) | 0.18 (0.21) | 0.29 (0.10) | 0.15 (0.25) |
| **Knee extensors** |  |  |  |  |  |
| **L_f_** | 0.26 (0.13) | 0.02 (0.66) | 0.15 (0.27) | 0.02 (0.68) | 0.09 (0.41) |
| **L_f_:L_m_** | 0.30 (0.10) | 0.01 (0.84) | 0.07 (0.47) | 0.01 (0.83) | 0.00 (0.94) |
| **F_max_** | 0.17 (0.24) | 0.14 (0.28) | 0.11 (0.34) | ***0.52 (0.01)*** | 0.16 (0.25) |
| **V_m_** | 0.02 (0.69) | 0.39 (0.05) | ***0.42 (0.04)*** | ***0.97 (<0.01)*** | ***0.58 (0.01)*** |
| **L_m_** | 0.03 (0.60) | ***0.54 (0.01)*** | 0.21 (0.17) | 0.36 (0.06) | ***0.65 (<0.01)*** |
| **Ankle dorsiflexors** |  |  |  |  |  |
| **L_f_** | 0.00 (0.90) | 0.29 (0.10) | 0.28 (0.11) | 0.26 (0.12) | 0.22 (0.17) |
| **L_f_:L_m_** | 0.02 (0.68) | 0.01 (0.76) | 0.01 (0.79) | 0.01 (0.75) | 0.00 (0.92) |
| **F_max_** | 0.01 (0.76) | 0.04 (0.57) | 0.04 (0.57) | 0.08 (0.41) | 0.10 (0.38) |
| **V_m_** | 0.01 (0.78) | ***0.65 (<0.01)*** | ***0.46 (0.03)*** | ***0.65 (<0.01)*** | ***0.65 (<0.01)*** |
| **L_m_** | 0.00 (0.91) | ***0.59 (<0.01)*** | ***0.46 (0.04)*** | ***0.68 (<0.01)*** | ***0.53 (0.01)*** |
| **Ankle plantarflexors** |  |  |  |  |  |
| **L_f_** | 0.04 (0.56) | 0.34 (0.07) | 0.26 (0.13) | ***0.51 (0.02)*** | 0.33 (0.08) |
| **L_f_:L_m_** | 0.20 (0.19) | 0.01 (0.78) | 0.06 (0.50) | 0.01 (0.76) | 0.05 (0.52) |
| **F_max_** | 0.04 (0.59) | ***0.58 (0.01)*** | ***0.52 (0.01)*** | 0.37 (0.06) | ***0.64 (<0.01)*** |
| **V_m_** | 0.00 (0.85) | ***0.57 (0.01)*** | ***0.60 (<0.01)*** | ***0.83 (<0.01)*** | ***0.68 (<0.01)*** |
| **L_m_** | 0.00 (0.85) | ***0.43 (0.03)*** | 0.28 (0.11) | ***0.67 (<0.01)*** | ***0.47 (0.02)*** |
