# Supplementary material for: In vivo human lower limb muscle architecture dataset obtained using diffusion tensor imaging
Source: PLoS One. 2019 Oct 15;14(10):e0223531. doi: 10.1371/journal.pone.0223531 (PMC6793854; doi:10.1371/journal.pone.0223531)
Supplement: S12 Table — Muscle masses from our data were estimated from muscle volumes. Lf:Lm- Muscle fiber length muscle length ratio. PCSA- Physiological cross-sectional area. Fmax- estimated maximum isometric force. Sarcomere lengths used to estimate optimal fiber lengths were sourced from Ward et al., [3]. (DOCX) [file pone.0223531.s012.docx]

| **Muscle** | **Muscle Mass (g)** | **Muscle length (mm)** | **Optimal fiber length (mm)** | **L_f_:L_m_** | **Pennation angle (°)** | **PCSA (mm^2^)** | **F_max_ (N)** | **F_max_ (%BW)** |
| --- | --- | --- | --- | --- | --- | --- | --- | --- |
| **Adductor magnus** | 274 (84%) | -76 (-20%) | 87 (60%) | 0.35 (89%) | -3 (-21%) | 474 (23%) | 142 (23%) | 30 (40%) |
| **Adductor longus** | 93 (124%) | 0 (0%) | 2 (2%) | 0.02 (3%) | 5 (69%) | 820 (126%) | 246 (126%) | 36 (149%) |
| **Adductor brevis** | 43 (79%) | -3 (-2%) | -27 (-26%) | -0.17 (-24%) | 5 (88%) | 768 (154%) | 230 (154%) | 34 (184%) |
| **Gracilis** | 43 (82%) | 57 (20%) | -55 (-24%) | -0.29 (-36%) | -2 (-22%) | 311 (142%) | 93 (142%) | 14 (177%) |
| **Semimembranosus** | 124 (92%) | -22 (-7%) | 89 (129%) | 0.34 (141%) | -3 (-21%) | -279 (-15%) | -84 (-15%) | -4 (-6%) |
| **Semitendinosus** | 97 (97%) | 28 (9%) | -10 (-5%) | -0.08 (-12%) | -5 (-40%) | 593 (123%) | 178 (123%) | 25 (143%) |
| **Biceps femoris- long head** | 91 (81%) | -87 (-25%) | 107 (109%) | 0.50 (178%) | 0 (-3%) | -132 (-12%) | -40 (-12%) | -3 (-7%) |
| **Biceps femoris- short head** | 37 (62%) | 55 (25%) | -1 (-1%) | -0.08 (-16%) | -3 (-24%) | 339 (66%) | 102 (66%) | 16 (85%) |
| **Sartorius** | 72 (92%) | 56 (12%) | 5 (1%) | -0.04 (-4%) | N/A | 159 (84%) | 48 (84%) | 7 (99%) |
| **Rectus femoris** | 152 (138%) | -40 (-11%) | 66 (87%) | 0.23 (109%) | -6 (-41%) | 503 (37%) | 151 (37%) | 28 (57%) |
| **Vastus lateralis** | 264 (70%) | 62 (23%) | 97 (97%) | 0.20 (52%) | -3 (-17%) | -304 (-9%) | -91 (-9%) | -1 (-1%) |
| **Vastus medialis** | 199 (83%) | -103 (-23%) | 62 (64%) | 0.25 (116%) | -16 (-53%) | 647 (31%) | 194 (31%) | 34 (44%) |
| **Vastus intermedius** | 378 (220%) | -59 (-14%) | 82 (83%) | 0.27 (111%) | 7 (155%) | 1268 (76%) | 380 (76%) | 60 (97%) |
| **Tibialis anterior** | 56 (70%) | 40 (16%) | 69 (101%) | 0.20 (72%) | -3 (-26%) | -135 (-12%) | -41 (-12%) | -1 (-2%) |
| **Extensor digitorum longus** | 39 (96%) | 58 (20%) | 69 (100%) | 0.16 (68%) | -3 (-31%) | 10 (2%) | 3 (2%) | 3 (15%) |
| **Extensor hallucis longus** | 1 (4%) | -4 (-2%) | 31 (42%) | 0.14 (45%) | -3 (-30%) | -74 (-27%) | -22 (-27%) | -2 (-20%) |
| **Medial gastrocnemius** | 130 (114%) | -16 (-6%) | 46 (89%) | 0.19 (102%) | 0 (2%) | 261 (12%) | 78 (12%) | 19 (24%) |
| **Lateral gastrocnemius** | 73 (117%) | 17 (7%) | 63 (107%) | 0.24 (87%) | -3 (-25%) | 189 (19%) | 57 (19%) | 11 (31%) |
| **Soleus** | 211 (76%) | -56 (-14%) | 102 (233%) | 0.30 (273%) | -17 (-58%) | -1954 (-38%) | -586 (-38%) | -61 (-32%) |
| **Mean** | **125 (94%)** | **-5 (<1%)** | **46 (66%)** | **0.14 (71%)** | **-3 (-5%)** | **182 (41%)** | **55 (41%)** | **13 (57%)** |
| **Hip adductors** | **113 (93%)** | **-5 (<1%)** | **2 (3%)** | **-0.02 (8%)** | **1 (28%)** | **593 (111%)** | **178 (111%)** | **29 (137%)** |
| **Knee flexors** | **84 (85%)** | **6 (3%)** | **38 (47%)** | **0.13 (57%)** | **-3 (-22%)** | **136 (49%)** | **41 (38%)** | **8 (63%)** |
| **Knee extensors** | **248 (128%)** | **-35 (-7%)** | **77 (83%)** | **0.24 (97%)** | **-4 (11%)** | **528 (34%)** | **159 (34%)** | **30 (49%)** |
| **Ankle dorsiflexors** | **32 (57%)** | **32 (11%)** | **56 (81%)** | **0.17 (62%)** | **-3 (29%)** | **-66 (-13%)** | **-20 (-13%)** | **<1 (-3%)** |
| **Ankle plantarflexors** | **138 (103%)** | **-18 (-4%)** | **70 (143%)** | **0.24 (154%)** | **-6 (-27%)** | **-501 (-2%)** | **-150 (-2%)** | **-10 (-8%)** |
