# Supplementary figures and images for: In vivo human lower limb muscle architecture dataset obtained using diffusion tensor imaging
Source: PLoS One. 2019 Oct 15;14(10):e0223531. doi: 10.1371/journal.pone.0223531 (PMC6793854; doi:10.1371/journal.pone.0223531)

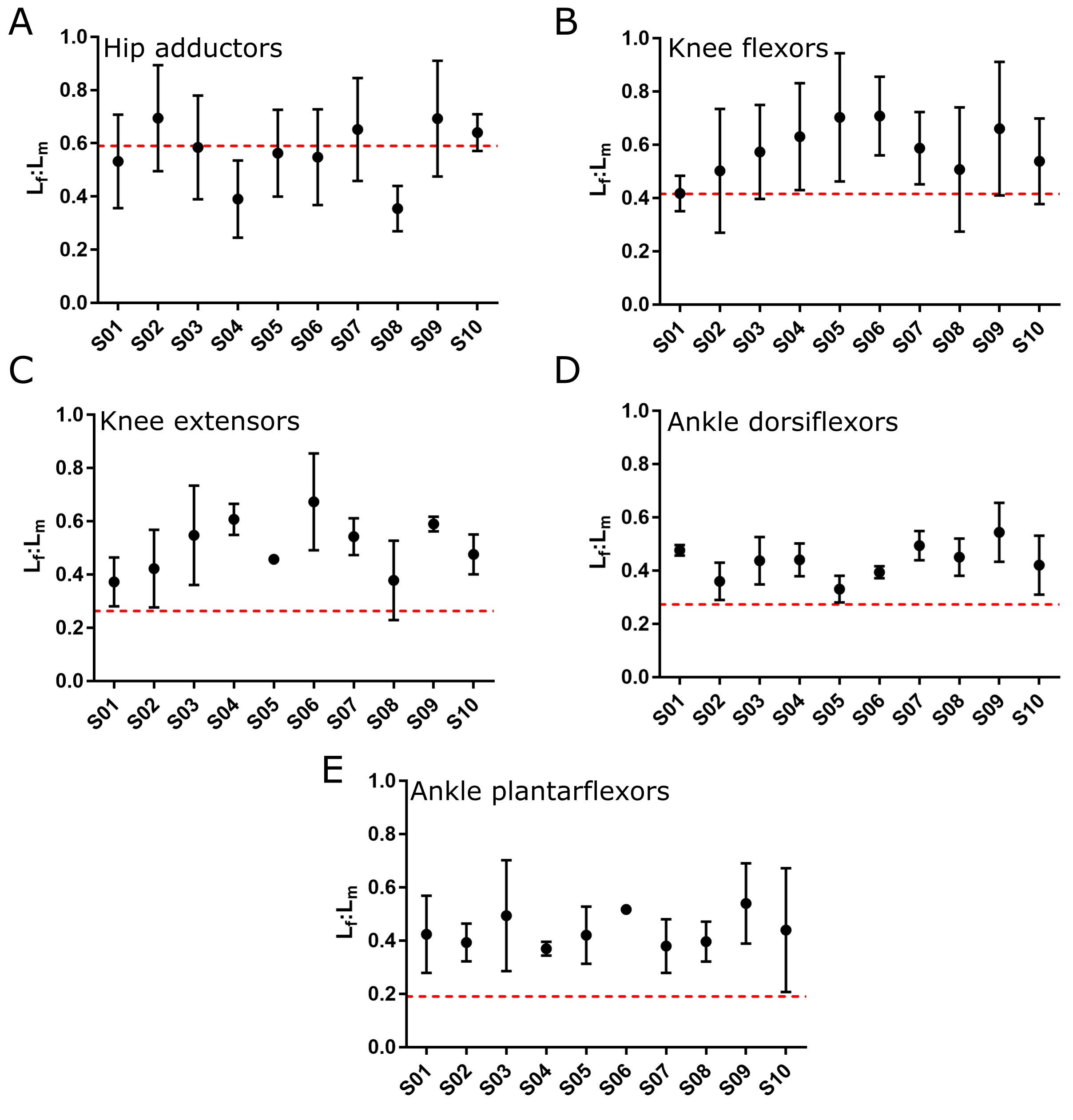

Supplement: S1 Fig — For muscle functional group classifications, see Table 1. Horizontal dashed line represents the mean value from Ward et al., [3]. (TIF) [file pone.0223531.s013.tif]

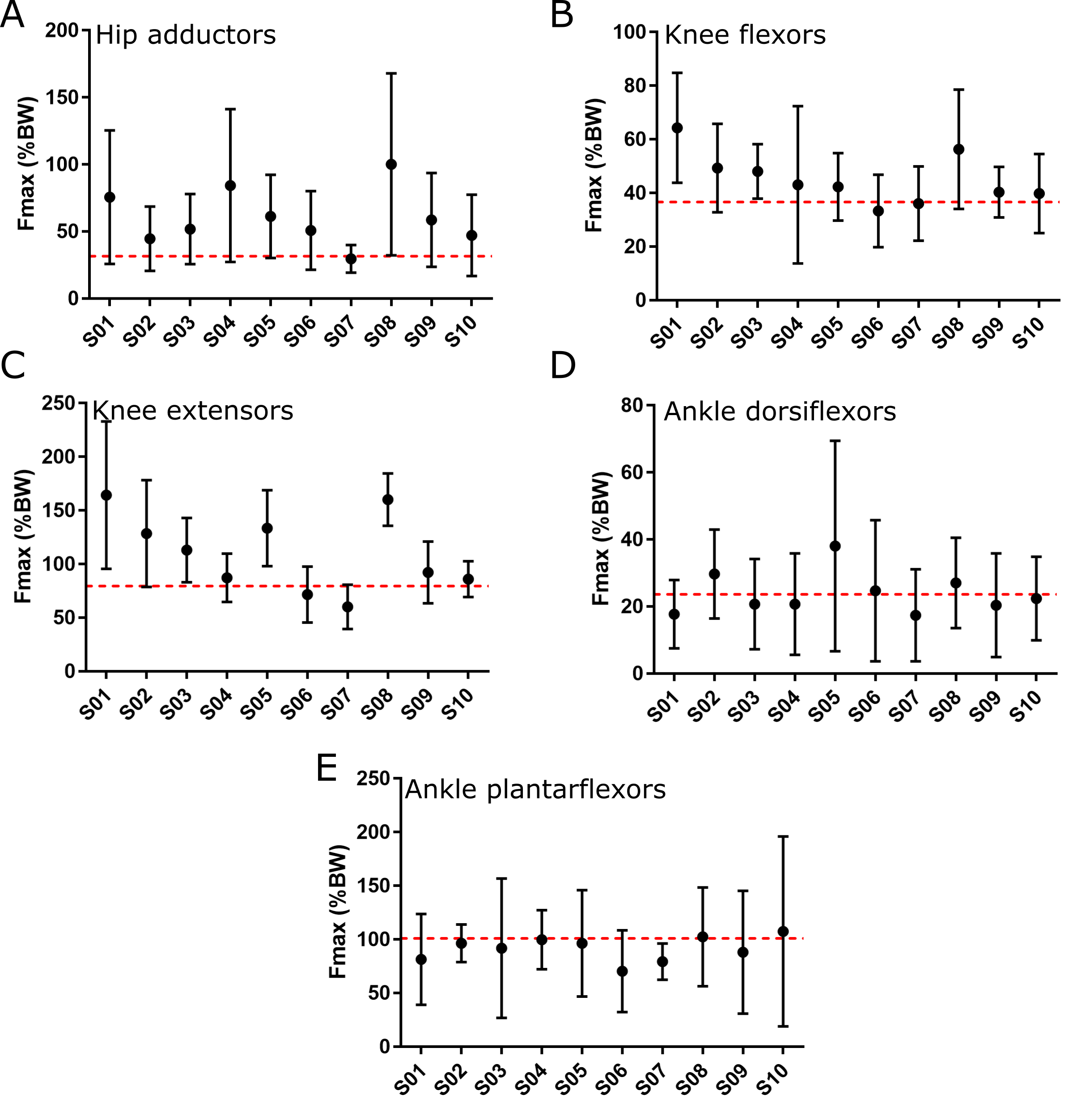

Supplement: S2 Fig — For muscle functional group classifications, see Table 1. Horizontal dashed line represents the mean value from Ward et al., [3]. (TIF) [file pone.0223531.s014.tif]
