# Supplementary material for: In vivo human lower limb muscle architecture dataset obtained using diffusion tensor imaging
Source: PLoS One. 2019 Oct 15;14(10):e0223531. doi: 10.1371/journal.pone.0223531 (PMC6793854; doi:10.1371/journal.pone.0223531)
Supplement: S1 File — (DOCX) [file pone.0223531.s015.docx]

***Comparison to previous architecture data***

To facilitate a direct comparison of the data presented here and previous data from cadaveric dissections detailed by Ward et al., [3], muscle masses were estimated from the MRI derived volumetric muscle meshes:

M_m_= V_m_ * $\text{ρ}$,

where M_m_ is muscle (belly) mass (g), V_m_ is muscle (belly) volume (mm^3^) and $\text{ρ}$ is the density of mammalian skeletal muscle (0.001056 gmm^-3^).

S12 Table lists the absolute and % differences of the present architecture data gathered from MRI, compared to previously reported cadaveric architecture data [3]. S1 and S2 Figs show mean L_f_:L_m_ and F_max_ values for each functional group, compared to mean values reported by Ward et al., [3].
